# Blocking IL-17A enhances tumor response to anti-PD-1 immunotherapy in microsatellite stable colorectal cancer

**DOI:** 10.1136/jitc-2020-001895

**Published:** 2021-01-17

**Authors:** Chao Liu, Ruiqi Liu, Bojun Wang, Jie Lian, Yang Yao, Haoxiu Sun, Chunhui Zhang, Lin Fang, Xin Guan, Jiaqi Shi, Shuling Han, Fei Zhan, Shengnan Luo, Yuanfei Yao, Tongsen Zheng, Yanqiao Zhang

**Affiliations:** 1 Department of Gastrointestinal Medical Oncology, Harbin Medical University Cancer Hospital, Harbin, China; 2 Translational Medicine Research and Cooperation Center of Northern China, Heilongjiang Academy of Medical Sciences, Harbin, China; 3 Department of Radiation Oncology, Sun Yat-sen University Cancer Center, Guangzhou, China; 4 School of Life Science and Technology, Harbin Institute of Technology, Harbin, China; 5 Heilongjiang Cancer Institute, Harbin, China

**Keywords:** cytokines, drug therapy, combination, immunotherapy, gastrointestinal neoplasms

## Abstract

**Background:**

Immune checkpoint inhibitors (ICIs), including anti-PD-1 therapy, have limited efficacy in patients with microsatellite stable (MSS) colorectal cancer (CRC). Interleukin 17A (IL-17A) activity leads to a protumor microenvironment, dependent on its ability to induce the production of inflammatory mediators, mobilize myeloid cells and reshape the tumor environment. In the present study, we aimed to investigate the role of IL-17A in resistance to antitumor immunity and to explore the feasibility of anti-IL-17A combined with anti-PD-1 therapy in MSS CRC murine models.

**Methods:**

The expression of programmed cell death-ligand 1 (PD-L1) and its regulation by miR-15b-5p were investigated in MSS CRC cell lines and tissues. The effects of miR-15b-5p on tumorigenesis and anti-PD-1 treatment sensitivity were verified both in vitro and in colitis-associated cancer (CAC) and APC^min/+^ murine models. In vivo efficacy and mechanistic studies were conducted using antibodies targeting IL-17A and PD-1 in mice bearing subcutaneous CT26 and MC38 tumors.

**Results:**

Evaluation of clinical pathological specimens confirmed that *PD-L1* mRNA levels are associated with CD8+ T cell infiltration and better prognosis. miR-15b-5p was found to downregulate the expression of PD-L1 at the protein level, inhibit tumorigenesis and enhance anti-PD-1 sensitivity in CAC and APC^min/+^ CRC models. IL-17A led to high PD-L1 expression in CRC cells through regulating the P65/NRF1/miR-15b-5p axis. Combined IL-17A and PD-1 blockade had efficacy in CT26 and MC38 tumors, with more cytotoxic T lymphocytes cells and fewer myeloid-derived suppressor cells in tumors.

**Conclusions:**

IL-17A increases PD-L1 expression through the p65/NRF1/miR-15b-5p axis and promotes resistance to anti-PD-1 therapy. Blocking IL-17A improved the efficacy of anti-PD-1 therapy in MSS CRC murine models. IL-17A might serve as a therapeutic target to sensitize patients with MSS CRC to ICI therapy.

## BACKGROUND

Colorectal cancer (CRC) is the third most commonly diagnosed cancer and ranks as the second most frequent cause of cancer mortality worldwide.^1^ Immune checkpoint inhibitors (ICIs), especially anti-PD-1 therapy, have dramatically reshaped the landscape of cancer therapy in recent years.^2^ Unfortunately, the role of ICIs in CRC is limited to the microsatellite instability-high (MSI-H) tumors. For patients with microsatellite stability (MSS) CRC (approximately 90%), the response rate was only 5%–10%.^3–5^ A recent clinical report, the REGONIVO trial, demonstrated that Regorafenib combined with anti-PD-1 therapy could achieve an objective response rate of 33% in patients with MSS CRC.^6^ However, limited by the scale and insufficient evidence of clinical practice, there is still an urgent need to explain the resistance mechanisms and improve the efficacy of ICIs in MSS CRC.

The regulation of programmed cell death-ligand 1 (PD-L1) protein expression in tumors driven by interferon γ (IFN-γ) signals at the transcriptional level is considered to be the major reason for its predictive value in ICIs therapy in non-small cell lung carcinoma (NSCLC), gastric cancer, and gastroesophageal junction tumors.^7 8^ However, in CRC, PD-L1 protein expression was not found to be associated with the response to ICIs or survival in registration studies.^9^ This strongly suggests the heterogeneity of PD-L1 in the regulation mechanism in CRC cells, especially by post-transcriptional regulations and post-translational modifications (PTM), such as microRNA, protein glycosylation, phosphorylation, and ubiquitination.^10 11^ Recently, there has been increasing interest in the research exploring the predictive value of *PD-L1* mRNA expression for ICIs therapy and survival.^12 13^ Several studies have shown that measurement of *PD-L1* mRNA expression is comparable to measuring PD-L1 protein levels, both analytically and clinically, and even for melanoma samples, the mRNA level may be superior to the protein level in to predict the efficacy of ICIs.^14^ However, this predictive value of the *PD-L1* mRNA level in CRC remains unclear.

The T-helper (Th) 17 and interleukin 17 (IL-17) signatures were proven to be associated with poor prognosis in patients with CRC.^15^ IL-17A/interleukin 17 receptor A (IL-17RA) activates extracellular regulated kinase, p38 mitogen-activated protein kinase, and nuclear factor kappa B (NF-κB) signaling pathways within transformed enterocytes and promotes the tumorigenesis and angiogenesis.^16^ IL-17A released from γδT17 cells has also been confirmed to promote the recruitment of myeloid-derived suppressor cells (MDSCs) in mice colon tumors.^17^ Another study established that IL-17 signaling pathway can increase the immunosuppressive activity of regulatory T cells (Tregs), resulting in tumor growth and development.^18^ In the era of ICIs, researchers have been renewed their interest in the key role of IL-17A in immunotherapy, especially in CRC. A clinical analysis from Johns Hopkins University suggested that the activation of IL-17A signaling is related to the failure of anti-PD-1 therapy in patients with MSS CRC.^19^ Research from MD Anderson suggested a novel combinatorial strategy (eg, anti-IL-17A and anti-PD-1) to overcome resistance to ICIs in MSS CRC.^20^ Intriguingly, IL-17+ cells exist at a much higher frequency in MSS tumors than in MSI-H tumors among patients with CRC.^21^ Although growing evidence suggests that IL-17A activity might drive resistance to antitumor immunity and contribute to the therapeutic failure, there is still uncertainty as to whether blocking IL-17A could enhance the sensitivity to ICIs of MSS CRC.

In the current study, we hypothesized that IL-17A-mediated accumulation of PD-L1 at the post-transcriptional level would promote immune escape in MSS CRC. Blocking IL-17A might enhance tumor response to anti-PD-1 therapy in MSS CRC murine models. A significant benefit was observed from blocking IL-17A combined with anti-PD-1 therapy in the subcutaneous CT26 and MC38 models. Mechanistic studies in colitis-associated cancer (CAC) and APC^min/+^ CRC models revealed that PD-L1 levels were upregulated by IL-17A and miR-15b-5p at the post-transcriptional level, thereby suppressing the efficacy of ICIs. These results indicated that targeting IL-17A might improve the response to ICIs in MSS CRC.

## MATERIALS AND METHODS

### Patients and specimens

A CRC tissue microarray containing samples from 101 cases of colon cancer and paired adjacent noncancerous tissue was purchased from Superchip Biotech (HColA180Su17) (Shanghai, China). Additionally, 21 pairs of MSS colorectal tumors and adjacent colon tissues, 27 cases of colitis cancer tissues, and tissue samples from 160 cases of colorectal tumors were obtained from patients who underwent surgery at Harbin Medical University Cancer Hospital from January 2013 to January 2016. All patients in this study have provided written informed consent for the use of their specimens and information for future investigations according to guidelines of the ethics committee.

### Cell lines

The murine colon cancer cell line CT26 was purchased from the American Type Culture Collection (Manassas, Virginia, USA) and the murine colon cancer cell line MC38 was purchased from National Infrastructure of Cell Line Resource (Beijing, China). Both murine cell lines were cultured in RPMI-1640 medium (Gibco, California, USA) supplemented with 10% fetal bovine serum (FBS, Gibco) and 1% penicillin/streptomycin antibiotics (Gibco). Human colon epithelial cell lines FHC and NCM460 and human MSS CRC cell lines SW1116, HT29, SW480, and SW620 were obtained from the Cell Bank of the Type Culture Collection, Chinese Academy of Sciences (Shanghai, China). Cell lines FHC, NCM460, and HT29 were cultured in RPMI-1640 (Gibco) medium, SW1116 in F12K (Gibco) medium, and both SW480 and SW620 were cultured in L15 (Gibco) medium. All media for the human cell lines were supplemented with 10% FBS and 1% penicillin/streptomycin antibiotics at the same concentrations as for the murine cell lines. Cell lines in the logarithmic growth phase will be used for reagents (cytokine) treating tests or subcultured to maintain high cell viability. CT26, MC38, HT29, SW480, and SW620 are subcultured about every 2 days, and FHC, NCM460, and SW1116 are subcultured every 3 days. Experiments were carried out within 6 months after acquisition of the cell lines. All cell lines were validated by STR DNA fingerprinting. In addition, mycoplasma contamination was ruled out using a PCR-based method.

### Analysis of MSI status by immunohistochemical staining (IHC) and PCR

MSI status were analyzed by IHC and PCR, respectively, and only the samples with the same results of MSI status detected by the two methods would be included in this study. IHC for MMR proteins including MLH1, MSH2, MSH6, and PMS2 was performed with mouse or rabbit monoclonal antibodies. Loss of a MMR protein was defined as the absence of nuclear staining of tumor cells in the presence of positive nuclear staining in internal controls. Tumor loss of at least one MMR protein was collectively designated as dMMR (or MSI-H), and tumors with intact MMR protein expression designated as proficient MMR (pMMR or MSS).^21^

In addition, MSI status was determined by PCR analysis using a 3730 sequencer (Life Technologies, Carlsbad, California, USA). For this purpose, prepared formalin-fixed paraffin-embedded (FFPE) tissue were diluted to 20 ng/µL, respectively, followed by addition of 2.8 µL ddH_2_O, 4 µL 2.5× Buffer A, 2 µL 5× MSI Primer Mix, and 0.2 µL Taq DNA Polymerase I. PCR amplification was carried out as follows: predenaturation at 95°C for 5 min, followed by 30 cycles at 94°C for 30 s, 60°C for 1 min, 70°C for 1 min; and then final extension at 60°C for 30 min. Finally, the temperature was reduced to 15°C, and samples were centrifuged at 3000 rpm for 1 min. NR-21 and BAT26 were labeled with a blue fluorescent dye, NR-27 and BAT-25 with a green dye, and NR-24 and MONO-27 with a yellow dye. Tumors were termed MSI-H/dMMR if one or more markers showed instability and MSS/pMMR in case of no mutation.^22^

### T-cell coculture model

CD8+ T cells were sorted from BABL/c or C57BL/6J mouse spleens using flow cytometry and placed in RPMI-1640 medium supplemented with IL-2 (1000 U/mL). On day 1, the cells were plated at a density of 2×10^6^ per well in six-well plates and stimulated with mouse IFN-γ (1000 U/mL). On day 2, anti-CD3 antibody (50 ng/mL) and IL-2 (1000 U/mL) were added into the medium to promote T-cell activation. The culture medium with IL-2 (1000 U/mL) was changed every 3 days. CT26 or MC38 cells in logarithmic growth phase were transfected with miR-15b-5p mimics or miR-SC (scrambled control) controls for 36 hours. Stimulated CD8+ T cells were subsequently harvested and cocultured with the CT26 or MC38 cells at a 10:1 ratio for 24 hours. T cells and cell debris were removed by washing with phosphate-buffered saline (PBS), and living tumor cells were analyzed using crystal violet staining.

### In vivo efficacy studies

For the studies, 2×10^5^ CT26 cells were injected subcutaneously in the flank of BALB/c mice (n=9 per group) and 1×10^6^ MC38 cells were injected subcutaneously in the flank of C57BL/6J mice (n=9 per group). Once tumors reached 50–100 mm^3^ volume, each mouse treated with 100 µg/mouse of isotype, anti-IL-17A, or anti-PD-1 Abs every 2 days. Tumor growth and survival were assessed. Tumor size was determined using electronic calipers to measure the length and width and calculated by L×W^2^/2.

### Lentiviral and adeno-associated virus (AAV)

Mouse miR-15b-5p sequences were cloned into pLent-Puro-CMV-miR-15b-5p lentiviral vectors (Vigene Biosciences, Jinan, China). Thereafter, the lentiviral vectors were used to package the viral particles. Next, CT26 and MC38 cell lines were infected with this virus and both stably transformed overexpressing miR-15bp-5p cell and mock infected cells selected with puromycin (1 µg/mL, Sigma-Aldrich, Missouri, USA).

AAV-miR-15b-5p sponge virus particles were packaged by Vigenebio (Jinan, China). The AAV infection of colon epithelial cells was based on a previously published method.^23^ After 12 hours of fasting, the mice were given an enema of PBS with AAVs. Under anesthesia, a soft catheter was inserted into the mouse anus at a depth of 4 cm, and 0.2 mL of PBS with AAV-miR-15b-5p sponge or AAV-vector (5×10^10^ vg/mL) was instilled into the mouse colon. After recovery from the anesthesia, water and food were provided.

### Murine models of colon cancer

BALB/c (female, 6 weeks old) or C57BL/6 mice (female, 6 weeks old) were purchased from the Vital River Laboratory (Beijing, China). C57BL/6J-Apc^Min/+^ mice (male, 6 weeks old) were purchased from the Model Animal Resource Information Platform (Nanjing, China). All animal studies were conducted with the approval of the Harbin Medical University’s Institutional Animal Care and Use Committee, in compliance with the ARRIVE guidelines for the care and use of laboratory animals. All mice were bred and maintained in-house on a regulated 12 hour day/night cycle.

CAC model, established as based on our previously published protocol, was used in this study.^24^ Briefly, BALB/c female mice were treated with two drugs: azoxymethane (AOM) and dextran sodium sulfate (DSS), to induce colon tumors. At 6 weeks of age, female BALB/c mice (n=40) were divided into control and blocking miR-15b-5p groups (n=20 per group). The experimental groups were injected intraperitoneally with 12.5 mg/kg AOM, after which they were given 2.5% DSS in water for 5 days, and then water only for 14 days. This cycle was repeated three times. Control groups were either untreated or they were treated with AOM or DSS only. Mice in all groups were sacrificed on day 100, including those with significant weight loss in AOM/DSS group.

C57BL/6J-Apc^Min/+^ mice (n=20) were divided into control and blocking miR-15b-5p groups (n=10 per group). Two weeks after administration of AAV, all the Apc^min/+^ mice and control mice were placed on a high 60% kcal diet (D12492, Xietong, Nanjing, China) to increase tumor development.^25^

### Public data collection and analysis

The gene expression data with standard annotation were downloaded from the cancer genome atlas (TCGA, https://portal.gdc.cancer.gov/) and uploaded to the CIBERSORT web portal (http://cibersort.stanford.edu/), and the algorithm was run using the LM22 signature.^26^ The GSE68306 data were downloaded from the website (https://www.ncbi.nlm.nih.gov/geo/query/acc.cgi?acc=GSE68306). Heatmaps were constructed and produced using R package.

### Statistical analysis

Statistical analyses of the immunohistochemistry results are explained in the online supplemental data section. For experiments with cell lines, the results are expressed as mean±SD and the statistical significance was assessed by two-tailed unpaired Student’s t test using GraphPad Instat3. Differences were considered significant when p<0.05. In the figures, * indicates p<0.05, **p<0.01, and ***p<0.001. For multiple comparisons, analysis of variance followed by Tukey’s multiple comparison test (when all groups were compared with each other) was applied. Cumulative survival time was estimated by the Kaplan-Meier method, and the log-rank test was applied to compare the groups. p<0.05 was considered statistically significant. P values and R values were calculated based on the analysis of Pearson’s correlation.

10.1136/jitc-2020-001895.supp1 Supplementary data

Detailed methods are enclosed in the online supplemental methods section.

## RESULTS

### *PD-L1* mRNA level is associated with CD8+ T cell infiltration and prognosis in MSS CRC

**Figure 1:**
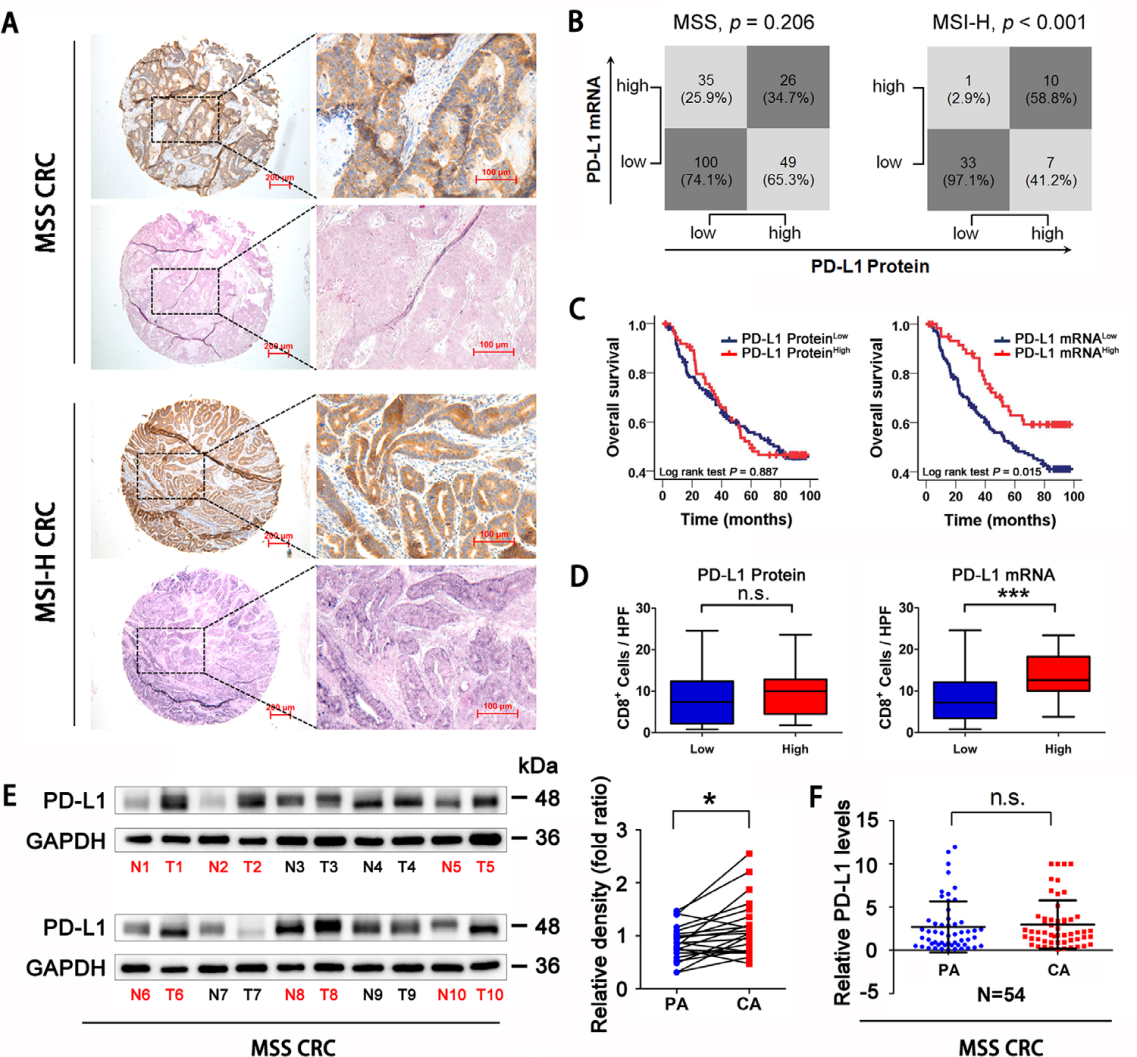
PD-L1 mRNA level is associated with CD8+ T cells infiltration and prognosis in MSS CRC. (A) Representative expression of PD-L1 protein and mRNA in MSS and MSI-H CRC tissues (scale bar, 100 µm; magnification scale bar, 200 µm). (B) Statistical analysis was conducted based on the level of PD-L1 protein and mRNA in patients with MSS and MSI-H. (C) Kaplan-Meier analysis of the overall survival rate of patients with MSS CRC, according to PD-L1 protein expression (left panel) and PD-L1 mRNA level (right panel). (D) Correlations of CD8+ cells densities with PD-L1 expression, according to PD-L1 protein expression (left panel) and PD-L1 mRNA level (right panel) in patients with MSS CRC (n=123). (E) Western Blotting detection showed that PD-L1 protein expression in MSS CRC tissues was higher than that in adjacent tissues (n=22). (F) RT-qPCR analysis showed that there was no significant difference in PD-L1 mRNA levels between MSS CRC tissues and adjacent tissues (n=54). P values and R values were calculated based on the analysis of Pearson’s correlation. The significance of survival difference was determined by the log rank test. Student’s t test. *P<0.05, **p<0.01, and ***p<0.001. CRC, colorectal cancer; MSI-H, microsatellite instability-high; MSS, microsatellite stable; PD-L1, programmed cell death ligand 1.

PD-L1 expression at the protein and mRNA levels was assessed in CRC (MSS type, n=210, MSI-H type, n=51; online supplemental table 1) tissues using IHC and in situ hybridization. The expression level (intensity) was scored as 0 (−, absent), 1 (+, weak), 2 (++, moderate) or 3 (+++, strong) (online supplemental figure 1A, B). Tissues with scores of 0 and 1 were divided into the low expression group, and tissues with scores of 2 and 3 were placed into high expression group. PD-L1 protein levels were consistent with mRNA levels only in MSI-H CRC tissues but not in MSS CRC (MSS CRC, p=0.206; MSI-H CRC, p<0.001; figure 1A, B). In particular, in the PD-L1 protein overexpression group of patients with MSS CRC, nearly 2/3 (65.3%) tissues had no elevation of the *PD-L1* mRNA level (figure 1B). Associations of PD-L1 protein or mRNA with prognosis and CD8 +cell infiltration were also analyzed in patients with MSS CRC. The results showed that patients with higher *PD-L1* mRNA levels had a better prognosis (Log-rank test p=0.015, figure 1C). In addition, CD8+ cell infiltration was increased in patients with high *PD-L1* mRNA (p<0.001, figure 1D). Neither overall survival nor CD8+ cell infiltration was significantly associated with PD-L1 protein status (figure 1C, D). CIBERSORT algorithm was used to analyze the composition of CD8 T cell, activated CD4 T cell, macrophages and regulatory T cell in CRC samples in TCGA database. We found that elevated *PD-L1* mRNA expression predicted better antitumor immune cell infiltration both in total CRC samples and in MSS CRC samples (online supplemental figure 1C, D). Compared with adjacent tissues of CRC, PD-L1 protein levels were elevated in MSS CRC tissues, but the difference in mRNA levels was not established (Protein p<0.05, mRNA p=0.621, figure 1E, F). Overall, PD-L1 mRNA expression level, but not the protein level, was associated significantly with CD8+ cell infiltration and patient survival prognosis in patients with MSS CRC.

10.1136/jitc-2020-001895.supp2 Supplementary data

10.1136/jitc-2020-001895.supp3 Supplementary data

10.1136/jitc-2020-001895.supp8 Supplementary data

### miR-15b-5p is a regulator of *PD-L1* at post-transcriptional level in MSS CRC

**Figure 2:**
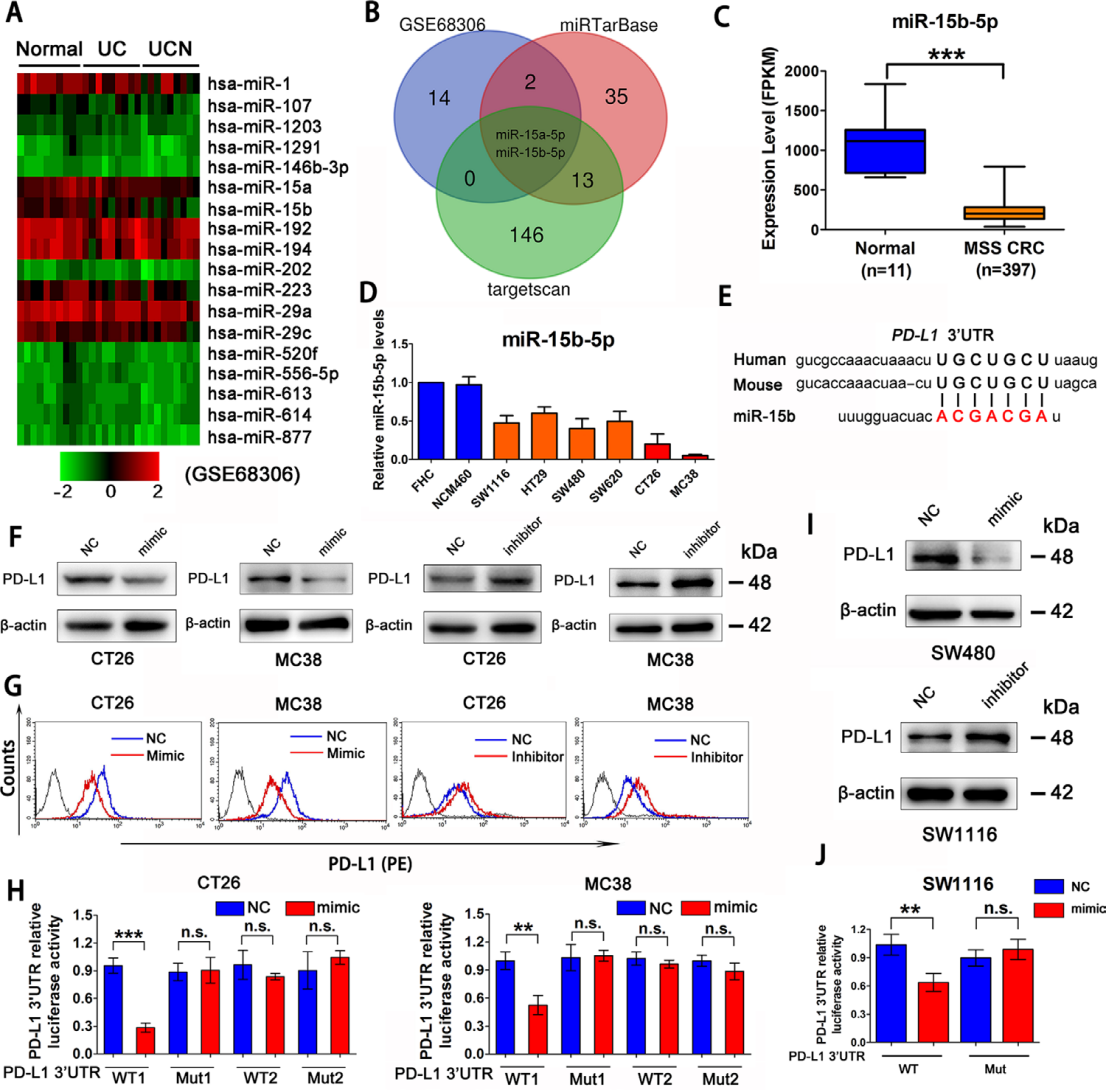
miR-15b-5p is a regulator of PD-L1 at post-transcriptional level in MSS CRC. (A) Heat map analysis of miRNA that gradually decreased from normal colon epithelial cells, to inflammation-associated colon epithelial cells, to cancer cells by GEO databases (GSE68306). (B) The Venn diagram indicates that miR-15b-5p, as a putative regulator of PD-L1 in CRC. (C) TCGA database analysis of miR-15b-5p expression decreased in MSS CRC samples compared with normal tissue. (D) RT-qPCR analysis that miR-15b-5p was downregulated in a plurality of MSS CRC cell lines compared with normal colon epithelial-derived FHC and NCM460 cell lines. (E) Schematic representation of predicted miR-15b-5p binding sites within the human and mouse PD-L1 3’-UTR. (F) Expression of PD-L1 in protein levels were analyzed by western blotting analysis in CT26 and MC38 cell lines treated with miR-15b-5p mimic or inhibitor. (G) PD-L1 expression on tumor cell surface was analyzed by flow cytometry in CT26 and MC38 cells after transfection of miR-15b-5p mimic or inhibitor. (H) Luciferase reporter activity was analyzed after cotransfection of miR-15b-5p mimic or negative control and WT PD-L1 3’-UTR luciferase reporter construct or mutant (Mut) construct into CT26 and MC38 cells. (I) Expression of PD-L1 in protein levels was analyzed by western blotting analysis after transfection of miR-15b-5p mimic or inhibitor into SW480 and SW1116 cell lines. (J) Luciferase reporter activity was analyzed after cotransfection of miR-15b-5p mimic or negative control and WT PD-L1 3’-UTR luciferase reporter construct or mutant (Mut) construct into SW1116 cells. The mean±SD from three independent experiments is represented. Data represent mean±SD. Results are representative of at least three separate experiments. Student’s t test. *P<0.05, **p<0.01, and ***p<0.001. CRC, colorectal cancer; MSS, microsatellite stable; PD-L1, programmed cell death ligand 1; UTR, untranslated region; WT, wild type.

CAC is reportedly an MSS tumor.^27 28^ We observed that PD-L1 protein level is elevated gradually from normal colon epithelial cells, to inflammation-associated colon epithelial cells, to CAC cells in patients (online supplemental figure 2A). A seminal study has shown that the expression levels of a series of microRNAs (miRNAs) decreased gradually during this process (GSE68306) (figure 2A).^29^ Next, miRTarBase and targetscan databases were used to predict candidate miRNAs that target *PD-L1* in mice and human cells, which showed that miR-15a-5p and miR-15b-5p are potential miRNAs targeting *PD-L1* (figure 2B). Transfection of miRNA mimics into CT26 and MC38 cell lines indicated that only miR-15b-5p reduced the levels of PD-L1 protein (online supplemental figure 2B). TCGA database also showed that miR-15b-5p expression was significantly decreased in MSS CRC samples compared with that in normal colon tissues (figure 2C). Moreover, miR-15b-5p was found to be downregulated in human MSS-type colon cancer cell lines (SW1116, HT29, SW480, and SW620) and mice colon cancer cell lines (CT26 and MC38) relative to that in the normal colon epithelial-derived cell lines, FHC and NCM460 (figure 2D).

10.1136/jitc-2020-001895.supp4 Supplementary data

Next, we analyzed the human and mouse *PD-L1* mRNA sequences and found the homology region of miR-15b-5p in the 3'-untranslated region (UTR) (figure 2E). CT26 and MC38 cell lines were used to assess the effect of miR-15b-5p on the expression of *PD-L1*. Cells transfected with miR-15b-5p mimics or inhibitors were collected 48 hours after transfection. Results showed that miR-15b-5p mimics could reduce the PD-L1 protein level in whole cell lysates and on the cell surface (figure 2F, G). In contrast, an inhibitor of miR-15b-5p increased PD-L1 protein levels (figure 2F, G); however, no significant change in *PD-L1* expression was observed at the mRNA level (online supplemental figure 2C). Luciferase reporter assays were performed using reporter plasmids containing either the wild-type (WT) *PD-L1* 3’-UTR or mutant (Mut) *PD-L1* 3’-UTR (online supplemental figure 2D). Transfection of the WT reporter and miR-15b-5p mimic into CT26 and MC38 cells significantly inhibited the luciferase activity compared with that in the control, whereas luciferase activity was restored when the predicted 3’-UTR-binding sites were mutated (figure 2H). In addition, analysis of inhibition of PD-L1 protein levels by miR-15b-5p binding to the 3’-UTR was also confirmed in human MSS CRC cell lines (SW1116, HT29, SW480, and SW620) (figure 2I, J and online supplemental figure 2E–G). All cell lines were tested during the logarithmic phase, and samples were collected after transfection 48 hours. In short, we demonstrated that miR-15b-5p could decrease the level of PD-L1 protein without affecting the mRNA level in mouse and human MSS CRC cells.

### miR-15b-5p inhibits CRC tumorigenesis and sensitizes tumors to anti-PD-1 therapy by targeting *PD-L1* in murine models

**Figure 3:**
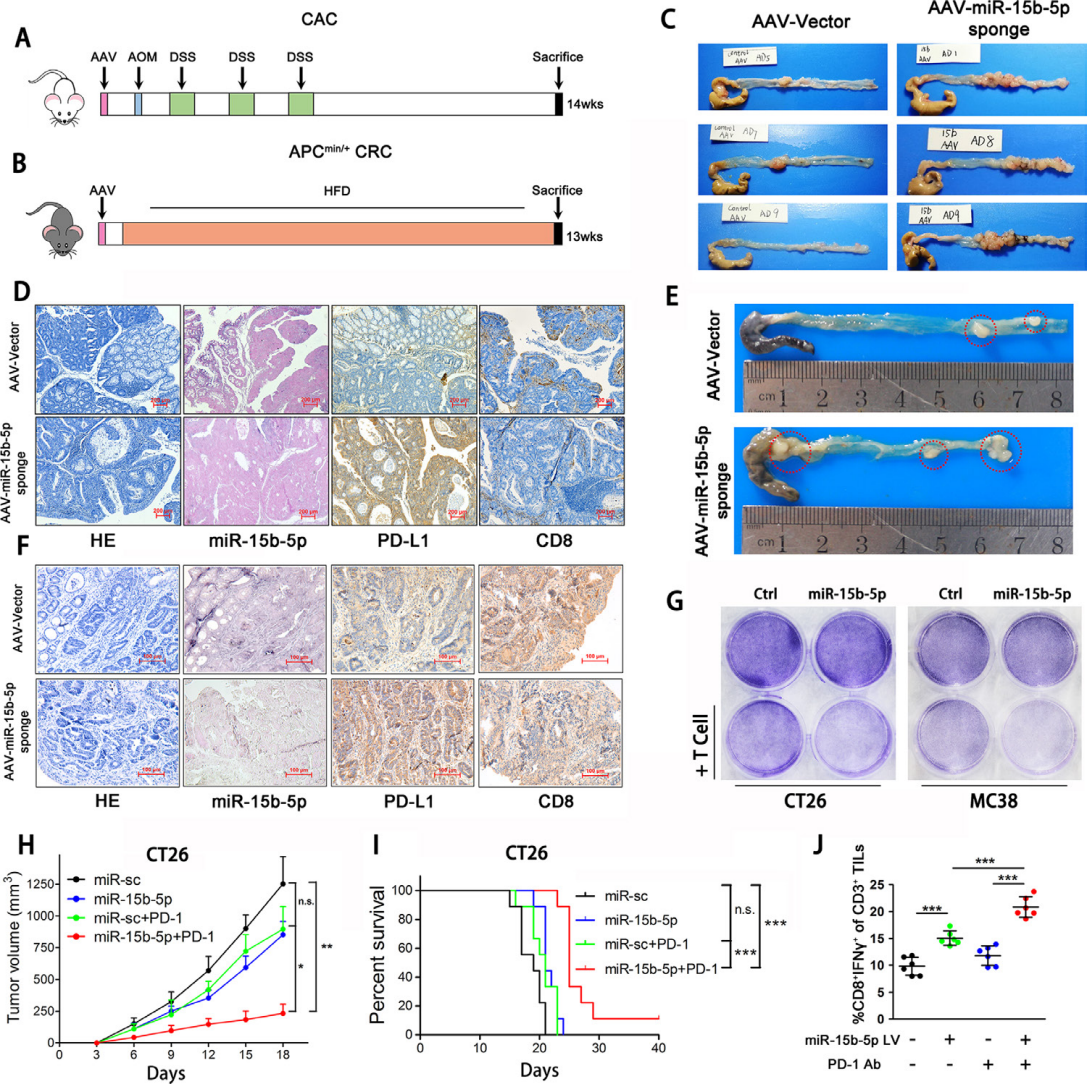
miR-15b-5p inhibits CRC tumorigenesis and sensitizes anti-PD-1 therapy by targeting PD-L1 in murine model. (A) Schematic representation of a mouse model of CAC following AAV (miR-15b-5p sponge) infection. (B) Schematic representation of a mouse model of APC^min/+^ colon cancer after infecting AAV (miR-15b-5p sponge). (C) Representative images were shown and observed colitis-associated tumor in the control and blocking miR-15b-5p group. (D) Representative images of H&E, miR-15b-5p, PD-L1, and CD8 +cells stained from CAC tumor tissues of control and blocking miR-15b-5p group mice (scale bar, 200 µm). (E) Representative images were shown and observed APC^min/+^ tumor in the control and blocking miR-15b-5p group. (F) Representative images of H&E, miR-15b-5p, PD-L1, and CD8+ cells stained from APC^min/+^ tumor tissues of control and blocking miR-15b-5p group mice (scale bar, 200 µm). (G) T cell-meditated tumor cell killing assay was performed in CT26 and MC38 cells infected with miR-15b-5p or control lentivirus. (H) Tumor growth of miR-sc, miR-15b-5p CT26 cells with or not PD-1 antibody treated in BALB/c mice (n=7 mice per group). (I) Survival of mice bearing syngeneic CT26 tumors following treatment with PD-1 antibody (n=9 mice per group). (J) Intracellular cytokine staining of CD8+ IFN-γ+ cells in the CD3+ T cell populations from isolated TILs. Data represent mean±SD. ANOVA followed by Tukey’s multiple comparison test was applied. Cumulative survival time was estimated by the Kaplan-Meier method, and the log-rank test was applied to compare the groups. *P<0.05, **p<0.01, and ***p<0.001. AAV, adeno-associated virus; ANOVA, analysis of variance; CAC, colitis-associated cancer; CRC, colorectal cancer; IFN, interferon; PD-L1, programmed cell death ligand 1; TIL, tumor-infiltrating lymphocyte.

To further validate the role of miR-15b-5p in PD-L1-mediated immunosuppression, we infected mice colons with AAV carrying an antisense sequence (miR-15b-5p sponge vector) to inhibit the miR-15b-5p expression and evaluated the effects of miR-15b-5p on tumorigenesis in CAC and APC^min/+^ colon cancer murine models (figure 3A, B). Blocking miR-15b-5p promoted tumorigenesis in the CAC model (figure 3C, online supplemental figure 3A–C). Compared with control group, the expression of PD-L1 was increased and CD8+ cell numbers were significantly decreased in the miR-15b-5p blocking group (figure 3D). Similar results were observed in the APC^min/+^ colon cancer model. After blocking miR-15b-5p, tumor progression in APC^min/+^ mice was more advanced (figure 3E). Meanwhile, PD-L1 expression and CD8+ cell depletion increased (figure 3F).

10.1136/jitc-2020-001895.supp5 Supplementary data

Next, we performed the T cell-mediated cancer cell killing and found that CT26 and MC38 cells overexpressing miR-15b-5p were more susceptible to T cell killing (figure 3G). CT26 (derived from BALB/c) and MC38 (derived from C57BL/6J) cells overexpressing miR-15b-5p were inoculated into mice, respectively (online supplemental figure 3D, E). Treatment with anti-PD-1 antibody reduced the growth rate of miR-15b-5p overexpressing tumors (figure 3H, online supplemental figure 3F) and prolonged the survival of both mouse strains (figure 3I, online supplemental figure 3G). Cytotoxic T lymphocyte cells (CTLs, CD3+ CD8+ IFNγ+) played the major antitumor effect in tumor milieu. And the increase in CTLs was often regarded as the main indicator of optimistic efficacy in the using of ICIs in CRC.^30 31^ We performed tumor infiltrating lymphocyte (TIL) analysis, and the results showed that the proportion of CTLs increased significantly in the miR-15b-5p overexpression with PD-1 antibody-treated group (figure 3J, online supplemental figure 3H). Above all, these results showed that blocking miR-15b-5p promotes CRC tumorigenesis by elevating PD-L1 levels and that overexpressing miR-15b-5p sensitizes tumors to anti-PD-1 therapy in murine models. Increasing miR-15b-5p expression might bring benefit for ICIs therapy; therefore, we continued to explore the mechanism of the decreased expression of miR-15b-5p in MSS CRC cells.

### IL-17A downregulates miR-15b-5p and enhances PD-L1 protein levels in MSS CRC cells

**Figure 4:**
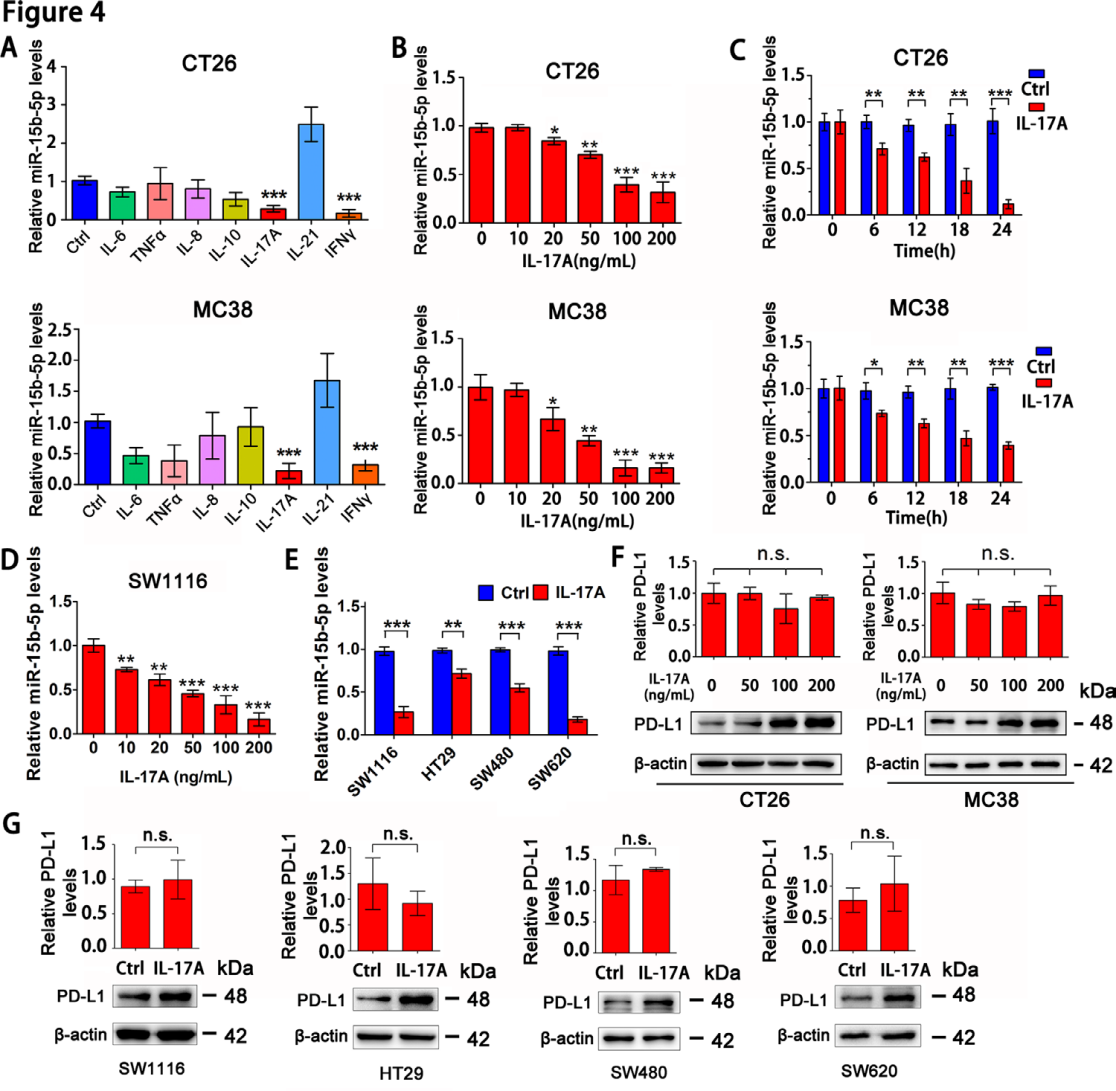
IL-17A downregulates miR-15b-5p and enhances PD-L1 protein expression in MSS CRC cells. (A) RT-qPCR was used to analyze the effects of seven cytokines on the expression of miR-15b-5p in CT26 and MC38 cells. (B) RT-qPCR was performed to determine the expression of miR-15b-5p in CT26 and MC38 cells treated with IL-17A at indicated concentration for 12 hours. (C) RT-qPCR was performed to determine the expression of miR-15b-5p in CT26 and MC38 cells treated with IL-17A (100 ng/mL) for the indicated times. (D) SW1116 cells were treated with IL-17A in a dose-dependent manner for 12 hours and then the expression of miR-15b-5p was detected. (E) Four human MSS CRC cell lines were treated with human IL-17A (100 ng/mL), the expression of miR-15b-5p was detected by RT-qPCR. (F) CT26 and MC38 cell lines were treated with different concentrations of IL-17A, protein and mRNA levels of PD-L1 were detected by Western Blotting and RT-qPCR, respectively. (G) SW1116, HT29, SW480, and SW620 cell lines were treated with IL-17A (100 ng/mL), protein, and mRNA levels of PD-L1 were detected by Western Blotting and RT-qPCR, respectively. Data represent mean±SD. Student’s t test. *P<0.05, **p<0.01, and ***p<0.001. CRC, colorectal cancer; IL, interleukin; MSS, microsatellite stable; PD-L1, programmed cell death ligand 1.

Inflammatory cytokine networks are critical mediators to regulate gene and miRNA expression in tumor cells. To ascertain which inflammatory factors are involved in miR-15b-5p down-regulation and PD-L1 upregulation, we treated CT26 and MC38 with seven cytokines for 12 hours and then analyzed the miR-15b-5p expression in tumor cells. These seven cytokines including tumor necrosis factor-α (TNF-α), interleukin-6 (IL-6), interleukin-8 (IL-8), interleukin-10 (IL-10), interleukin-17A (IL-17A), interleukin-21 (IL-21), and IFN-γ are well-known core proinflammatory cytokines or immunomodulatory cytokines in CRC.^32^ Only IL-17A and IFN-γ significantly downregulated the miR-15b-5p level (figure 4A). IFN-γ, a cytokine associated with TH1 cell response, is not highly enriched in CRC.^33^ Therefore, we investigated the effects of IL-17A on miR-15b-5p expression. We found that the miR-15b-5p level decreased significantly in CT26 and MC38 in a time and dose-dependent manner after IL-17A treatment (figure 4B, C). Meanwhile, IL-17A could reduce the expression of miR-15b-5p in SW1116 cell line in a dose-dependent manner (figure 4D). Moreover, similar results were observed in other human MSS CRC cell lines (HT29, SW480, and SW620) treated with IL-17A (figure 4E).

In addition, we examined the effect of IL-17A on the expression of PD-L1 in CT26 and MC38 cell lines. The results showed that IL-17A could increase the PD-L1 protein level, but the mRNA level did not change significantly (figure 4F). This phenomenon was also observed in human colon cancer cell lines (SW1116, SW480, SW620, and HT29) (figure 4G). Collectively, these data suggested that IL-17A could reduce the expression of miR-15b-5p and increase the level of PD-L1 protein in MSS CRC cell lines.

### NRF1 is the major transcription factor for IL-17A to accumulate PD-L1 protein

**Figure 5:**
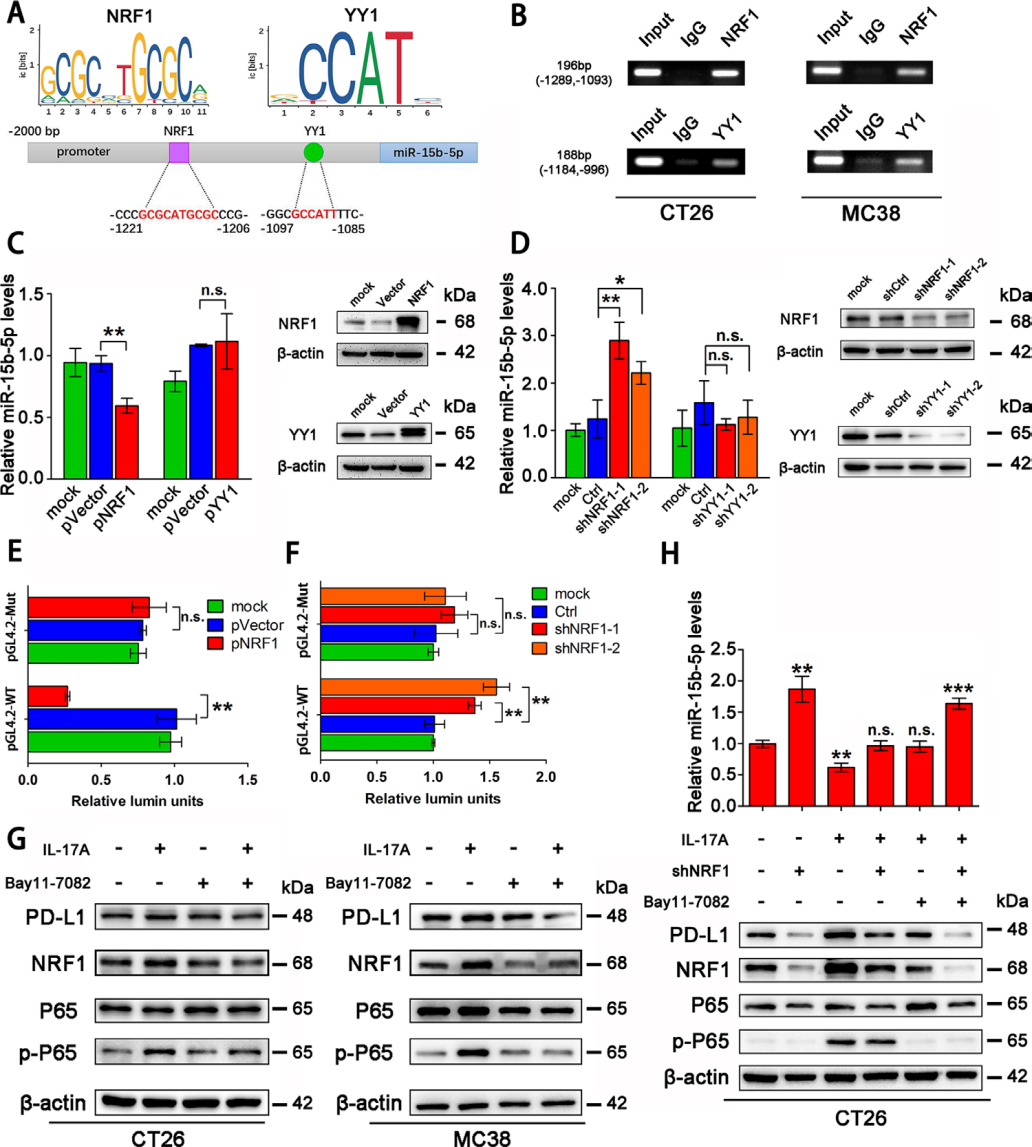
NRF1 is the major transcription factor for IL-17A to accumulate PD-L1 protein. (A) Schematic representation of predicted NRF1 and YY1 binding sites in the promoter of mouse miR-15b-5p. (B) Ch-IP analyzes of NRF1 or YY1 binding to the miR-15b-5p promoter by antibodies against NRF1 or YY1 in CT26 and MC38 cells. (C, D) Effects of NRF1/YY1 overexpression or knockdown on miR-15b-5p expression through RT-qPCR analysis. (E, F) Modulation of the miR-15b-5p promoter activity by NRF1 overexpression or knockdown in CT26 cells. (G) The regulation of IL-17A/P65 signaling on NRF1 and PD-L1 expression in CT26 and MC38 cells. (H) The effect of the IL-17A/P65/NRF1 axis on miR-15b-5p expression in CT26 cells. Data represent mean±SD. Results are representative of at least three separate experiments. Student’s t test. *P<0.05, **p<0.01, and ***p<0.001. Ch-IP, chromatin immunoprecipitation; NRF1, nuclear respiratory factor 1; PD-L1, programmed cell death ligand 1.

To investigate the mechanism by which IL-17A regulates miR-15b-5p and PD-L1 expression, we searched for potential transcription factor binding sites in the miR-15b-5p promoter region through TransFac programs and data from a previous study.^34^ We found that two transcription factors, nuclear respiratory factor 1 (NRF1) and Yin Yang 1 (YY1), were candidate transcription factors for miR-15b-5p (figure 5A). Interestingly, both transcription factors were confirmed to bind to their target motif in CT26 and MC38 by chromatin immunoprecipitation (Ch-IP) assay (figure 5B). NRF1 was also able to bind to the promoter region of miR-15b-5p in SW1116 and HT29 cell lines (online supplemental figure 4A). After overexpressing NRF1 and YY1 in CT26 and MC38 cells, respectively, we found that NRF1 could downregulate miR-15b-5p expression but YY1 could not, both in the two cell lines (figure 5C, online supplemental figure 4B). In addition, knockdown of *NRF1*, but not *YY1*, could upregulate miR-15b-5p level in cells (figure 5D, online supplemental figure 4C). Two pGL4.2 luciferase reporters were constructed, WT and mutant (Mut) miR-15b-5p promoter plasmids, to investigate the regulation by NRF1 of miR-15b-5p (online supplemental figure 4D). The relative activity of the pGL4.2-WT-reporter was reduced by *NRF1* overexpression and enhanced by knockdown of NRF1 in CT26 and MC38 cells (figure 5E, F, online supplemental figure 4E, F). However, the pGL4.2-Mut-reporter was not affected by NRF1 in either cell line (figure 5E, F, online supplemental figure 4E, F).

10.1136/jitc-2020-001895.supp6 Supplementary data

We further examined whether IL-17A represses miR-15b-5p via NRF1 in CRC cells. NF-κB (P65) has been reported to be able to activate NRF1; therefore, we treated CT26 and MC38 cells with IL-17A and the inhibitor of P65 (Bay11-7082), respectively. The results showed that IL-17A increased the expression of NRF1 and PD-L1 in both cell lines. When CT26 and MC38 cell lines were treated simultaneously with IL-17A and Bay11-7082, the expression levels of NRF1 and PD-L1 were restored to control levels (figure 5G). After knocking down *NRF1* in CT26, IL-17A was unable to increase the expression of miR-15b-5p and PD-L1 (figure 5H). Taken together, these results showed that IL-17A suppresses the level of miR-15b-5p through the P65/NRF1/miR-15b-5p signal axis, thus promoting the expression of PD-L1.

### IL-17A is elevated in MSS CRC and is associated with NRF1, miR-15b-5p, and PD-L1 expression in tumor tissues

**Figure 6:**
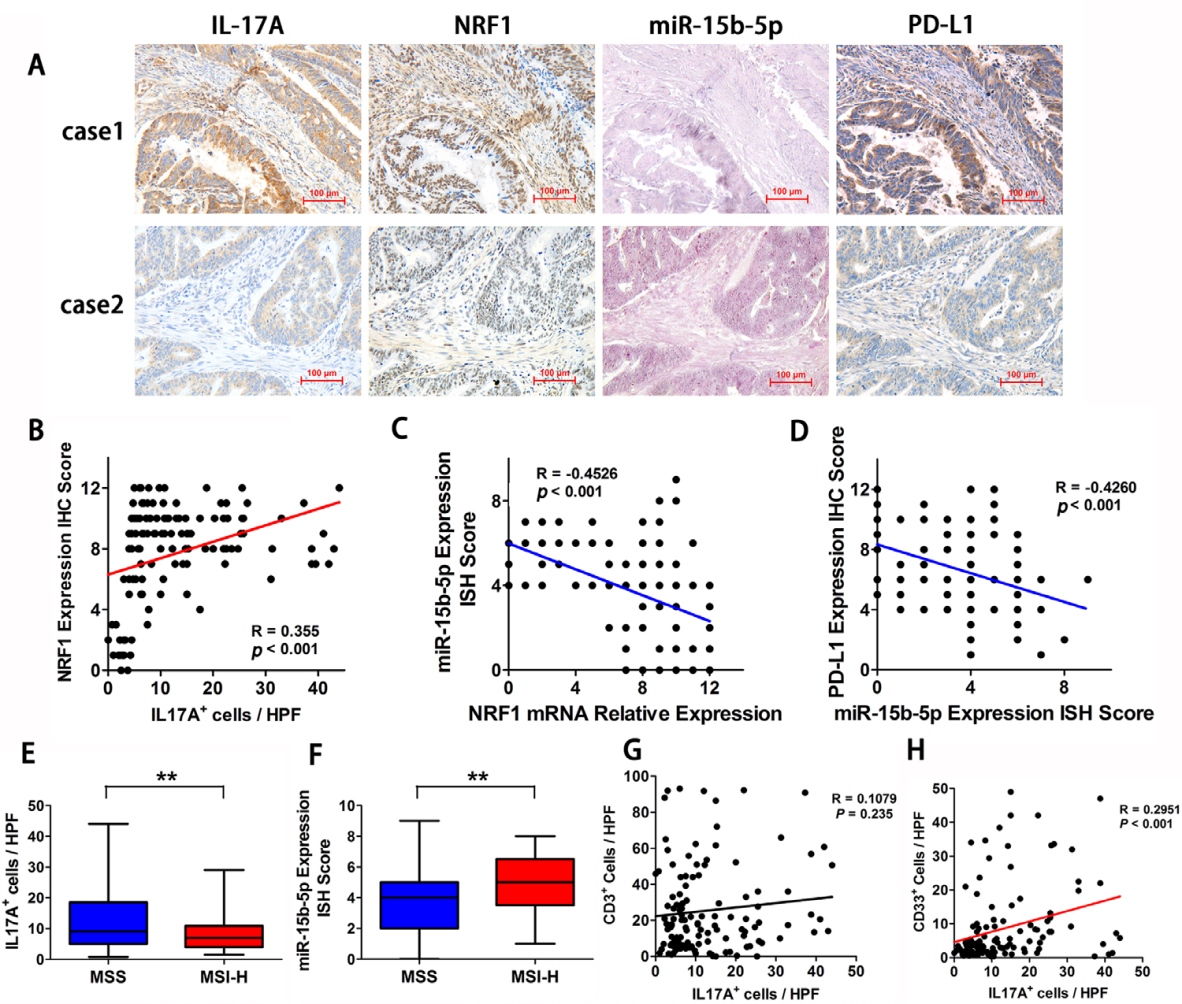
IL-17A is elevated in MSS CRC and is associated with NRF1, miR-15b-5p, and PD-L1 expression in tumor tissues. (A) Two representative IHC or ISH staining results for IL-17A, miR-15b-5p, NRF1, and PD-L1 in tissues of patients with CRC (scale bar, 100 µm). (B) IL-17A positive cell count was positively correlated with NRF1 expression (p<0.001, R=0.355). (C) NRF1 expression was negatively correlated with miR-15b-5p expression (p<0.001, R=−0.4526). (D) miR-15b-5p expression was negatively correlated with PD-L1 expression (p<0.001, R=−0.4260). (E) IL-17A positive cells is elevated in tumors of MSS relative to MSI-H tumors (p<0.01). (F) miR-15b-5p expression is decreased in tumors of MSS relative to MSI-H tumors (p<0.01). (G) Correlations between IL-17A positive cells and CD3+ cells is in MSS CRC tissues (p=0.1079, R=0.235). (H) Correlations between IL-17A positive cells and CD33 positive cells is in MSS CRC tissues (p<0.001, R=0.2951). P values and R values were calculated based on the analysis of Pearson’s correlation. Student’s t test. *P<0.05, **p<0.01, and ***p<0.001. CRC, colorectal cancer; IHC, immunohistochemical staining; ISH, in situ hybridization; IL, interleukin; MSI-H, microsatellite instability-high; MSS, microsatellite stable; NRF1, nuclear respiratory factor 1; PD-L1, programmed cell death ligand 1.

To validate the signal transmission in MSS CRC tissues, we analyzed the correlations between IL-17A, NRF1, miR-15b-5p, and PD-L1 expression in MSS CRC tumor specimens obtained from 160 patients. The results indicated that miR-15b-5p expression is inversely associated with NRF1, IL-17A, and PD-L1 (figure 6A). The statistical results showed that IL-17A+ cell count positively correlated with NRF1 expression (R=0.355, p<0.001; figure 6B), NRF1 expression inversely correlated with miR-15b-5p expression (R=−0.4526, p<0.001; figure 6C), and miR-15b-5p expression negatively correlated with PD-L1 expression (R=−0.4260, p<0.001; figure 6D). Next, we compared the expression levels of IL-17A and miR-15b-5p in tumor tissues between patients with MSS and patients with MSI-H CRC. We found that IL-17A positive cells were elevated in MSS tumors relative to those in MSI-H tumors (figure 6E). Moreover, miR-15b-5p expression was decreased in MSS tumors compared with that in MSI-H tumors (figure 6F). We further examined the correlation between the counts of CD3+ cell, MDSCs, and IL-17A expression. CD33 was selected as the marker of MDSCs. Surprisingly, there was no significant correlation between IL-17A+ cell and CD3+ cell counts in MSS CRC milieu (R=0.1079, p=0.235; figure 6G). In contrast, IL-17A+cell count correlated positively with the number of CD33+ cell in MSS CRC tissues (R=0.2951, p<0.001; figure 6H). The above results confirmed that IL-17A is elevated in MSS CRC and is associated with NRF1, miR-15b-5p, and PD-L1 expression in tumor tissues.

### Blocking IL-17A enhances the efficacy of anti-PD-1 therapy in murine model by promoting PD-L1 protein degradation

**Figure 7:**
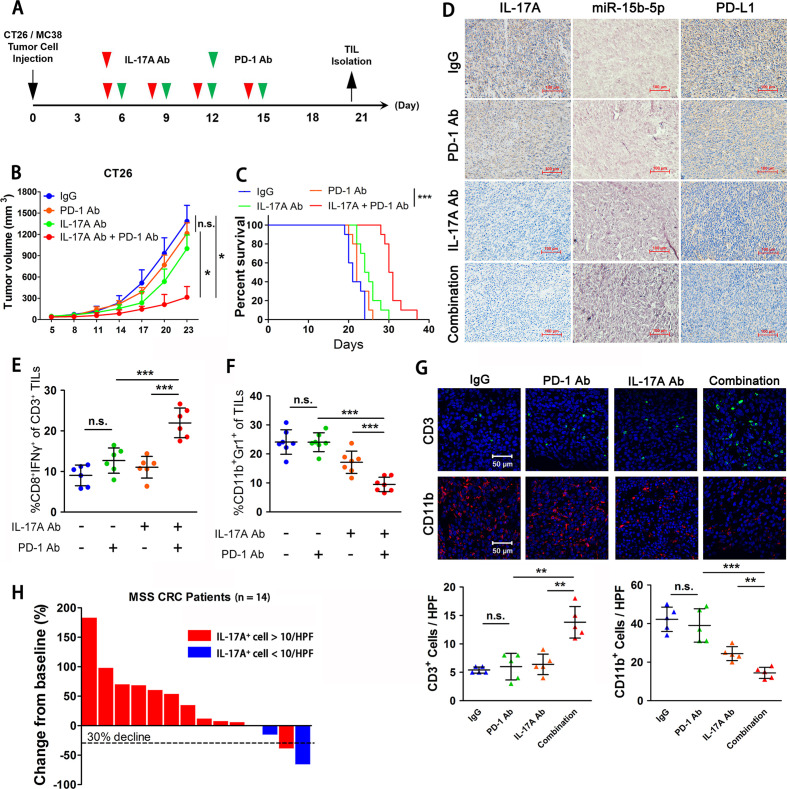
Blocking IL-17A enhances the efficacy of anti-PD-1 therapy in murine model by promoting PD-L1 protein degradation. (A) Schematic diagram illustrating the treatment protocol of IL-17A antibody and PD-1 antibody in mice. (B) Tumor growth of CT26 cells with IL-17A antibody and/or PD-1 antibody treated in BALB/c mice (n=7 mice per group). (C) Survival of mice bearing CT26 tumors following treatment with IL-17A antibody and/or PD-1 antibody (n=9 mice per group). (D) IHC analysis of IL-17A and PD-L1 expression, ISH analysis of miR-15b-5p expression in CT26 tumors of each group of mice. (E) FACS analysis of staining of CD8+ IFN-γ+ cells in the CD3+ T cell populations from isolated tumor-infiltrating lymphocytes. (F) FACS analysis of staining of CD11b+ and Gr1+ cells in the tumor-infiltrating lymphocyte. (G) Immunofluorescence was used to analyze the staining of CD3+ cells and CD11b+ cells in CT26 tumor mice. (H) Waterfall plot showing change in 14 patients with MSS CRC received anti-PD-1 therapy tumor volume compared with baseline before treatment. Data represent mean±SD. ANOVA followed by Tukey’s multiple comparison test was applied. Cumulative survival time was estimated by the Kaplan-Meier method, and the log-rank test was applied to compare the groups. *P<0.05, **p<0.01, and ***p<0.001. ANOVA, analysis of variance; CRC, colorectal cancer; FACS, fluorescence-activated cell sorter; IFN, interferon; IHC, immunohistochemical staining; IL, interleukin; ISH, in situ hybridization; MSS, microsatellite stable; PD-1: programmed death1; PD-L1, programmed cell death ligand 1.

All these above results indicated that blocking IL-17A/miR-15b-5p/PD-L1 signal axis had the potential to enhance the efficacy of anti-PD-1 therapy in MSS CRC. Therefore, we verified the effect of combined anti-IL-17A antibodies and anti-PD-1 antibodies in CT26 and MC38 tumor-bearing mice (figure 7A). The results demonstrated that the tumor growth in the combined group was significantly slower and the survival rate was also prolonged (figure 7B, C, online supplemental figure 5A, B). Meanwhile, we analyzed the expression of IL-17A, miR-15b-5p, and PD-L1 in each mouse tumor tissues and found that the expression of IL-17A and PD-L1 was decreased and the expression of miR-15b-5p was enhanced in the combined treatment group (figure 7D). The tumor-infiltrated activated CD8+ T cell (IFNγ+CD8+CD3+) population was increased in the combined treatment group (figure 7E and online supplemental figure 5C). In this xenograft experiment, we also found that MDSCs (Gr1 +CD11b+) numbers were reduced in tumors after blocking IL-17A (figure 7F and online supplemental figure 5D). Immunofluorescence analysis showed that in the combined treatment group, CD3+ cell numbers were markedly increased, while CD11+ cell numbers were reduced (figure 7G, online supplemental figure 5E). In summary, we confirmed the significant effect of anti-IL-17A combined with anti-PD-1 therapy in the MSS CRC murine model and observed the effect of antitumor immune response induced by this combination therapy in the tumor microenvironment.

10.1136/jitc-2020-001895.supp7 Supplementary data

To further demonstrate the potential of targeting the IL-17A signal in patients with MSS CRC, we collected 14 patients with MSS CRC treated with anti-PD-1 antibodies, and 2 of them achieved partial response (PR) after treatment. We then detected the IL-17A+cells in their tumor tissues and found that the counts of IL-17A+cells in two patients were less than 10/HPF, including one patient with PR and one patient with stable disease (SD, tumor reduction of 16% relative to baseline) (figure 7H). Therefore, this result suggested that IL-17A might serve as a promising therapeutic target for sensitizing ICIs therapy in patients with MSS CRC.

## DISCUSSION

PD-L1 expression, as assessed using IHC, failed to predict the efficacy of ICIs in CRC.^35^ Data from a number of recent studies have proposed that the level of *PD-L1* mRNA strongly correlated with prognosis and immune infiltration of tumors, especially in hepatocellular carcinoma (HCC), NSCLC, and malignant melanoma.^12–14^ To date, no study has investigated this predictive value of *PD-L1* mRNA in CRC. Herein, we examined PD-L1 expression in MSS and MSI-H tumor tissues from 261 patients with CRC at both the protein and mRNA levels. Notably, we confirmed that patients with MSS with elevated levels of *PD-L1* mRNA had a better prognosis and more CD8 +cell infiltration. Numerous clinical studies have also confirmed that the enrichment of CD8+ T cells indicates a good prognosis for patients with CRC and the potential of benefiting from ICIs treatment.^36–39^ Extraction of MSS CRC samples from TCGA database (mRNA expression data) also confirmed that high expression of *PD-L1* predicted a better antitumor immune cell infiltration. Conversely, the correlation between the PD-L1 protein level and CD8+ cell infiltration or overall survival was not observed in patients with MSS CRC. Thus, the *PD-L1* mRNA level, rather than the protein level, might be a potential biomarker to predicting prognosis and the efficacy of ICIs in patients with MSS CRC.

The expression and clinical significance of PD-L1 protein and mRNA is inconsistent in patients with MSS CRC, indicating that the regulation of PD-L1 occurs post-transcriptional. Recently, research studies explored the post-transcriptional regulations and PTM of PD-L1 expression and their effects on immunosuppression.^10 11^ In the current study, miR-15b-5p was identified and confirmed to inhibit PD-L1 expression in MSS CRC cells and murine models via post-transcriptional regulation. Consistent with our findings, other investigators have identified that miR-15b levels were significantly lower in the PD-L1-positive samples in malignant pleural mesothelioma.^40^ In addition, CAC and APC^min/+^ murine models further confirmed that blocking miR-15b-5p promoted CRC tumorigenesis by inhibiting the recruitment of CD8+ T cells. Significantly, overexpression of miR-15b-5p could enhance the efficacy of anti-PD-1 therapy in CT26 and MC38 tumors by increasing the counts of CTLs in tumors. Previous research has established that miR-15b plays an important role in the diagnosis, metastasis, and resistance of CRC.^41–43^ The results of the present study provided insights into the function of miR-15b-5p in reshaping the tumor immune microenvironment by downregulating PD-L1. At the PTM level, COP9 signalosome complex subunit 5 (CSN5) and CKLF Like MARVEL Transmembrane Domain Containing 6 (CMTM6) had been proven to lead to the accumulation of PD-L1 via the ubiquitin/proteasome pathway in CRC, which was meaningful for the activity of CD8+ T cells.^44 45^ Overall, our discovery of the effects of miR-15b-5p, combined with the PTM mediators of PD-L1, provided potential explanations for why the clinical significance of PD-L1 protein is not consistent with that of its mRNA in MSS CRC.

Interferons are not the only inflammatory stimuli that have been linked to PD-L1 expression. TNF-α, IL-6, IL-10, and IL-1β also have been shown to induce PD-L1.^10^ IL-17A is a new player in the CRC cytokine milieu, which is associated with tumorigenesis, angiogenesis, and metastasis of CRC.^16^ Several lines of evidence suggest that IL-17A can increase the expression of PD-L1 and promote tumor progression in HCC or ovarian cancer.^46 47^ Nevertheless, the detailed mechanism was not established. In this study, we observed that IL-17A could stimulate an increase in PD-L1 protein levels in CRC cells and tissues. In addition, we revealed that NRF1 is the key molecular mediator in this process and that IL-17A could enhance the expression of PD-L1 in CRC cells through the P65/NRF1/miR-15b-5p axis. To the best of our knowledge, this is the first report of the detailed mechanism by which IL-17A promotes PD-L1 expression in CRC cells. This regulatory relationship between IL-17A and PD-L1 indicates the potential of combining anti-IL-17A therapy to enhance the sensitivity of ICIs in MSS CRC.

Correlative evidence also suggested that IL-17 activity might drive escape from antitumor immunity and contribute to the therapeutic failure, especially in MSS CRC. First, the Th17/IL17A signature was found to be associated with poor prognosis in patients with CRC and Th17 cells existed in much higher frequency in MSS tumors than in MSI-H tumors.^15 21^ Second, recent clinical evidence from Johns Hopkins University suggested that patients with MSS CRC who express high levels of PD-L1, are infiltrated by CTLs, and have no IL-17 producing TILs, might have a better response to anti-PD-1 therapy.^19^ Third, to accelerate CRC progression, γδT cells reportedly promote the recruitment and expansion of MDSCs by releasing IL-17A.^17^ A recent report also confirmed that IL-17 signaling inhibits the production of CXCL9/10 chemokines to reduce the infiltration of CTLs and Tregs into the CRC milieu.^18^ However, few studies have investigated the feasibility of such a promising scheme of combination of targeting IL-17A and anti-PD-1, whether in a murine model or clinical trial. This prospective study was designed to confirm the effect of this combined scheme in the subcutaneous CT26 and MC38 tumors. Combined IL-17A and PD-1 blockade elicited significant efficacy and extended survival in the MSS CRC models. Another important observation was that anti-IL-17A and anti-PD-1 combination increased the numbers of CTLs (CD8+IFNγ+CD3+) and reduced those of MDSCs (CD11b+Gr1+) in tumors. This regimen clearly adjusts the phenotypic properties of the tumor immune environment and might make a subject more likely to respond to immunotherapy. Meanwhile, we performed further verification in 14 patients with MSS CRC treated with anti-PD-1 drugs, which also proved that patients with loss of IL-17A+cells were more likely to benefit from ICIs treatment.

Several potential limitations of this study should be noted. First, although numerous studies support the potential value of blocking IL-17A for antitumor therapy, some researchers are cautious about the dual role of Th17/IL-17A signaling in tumor progression.^48^ We believe that it is based on the specific immune landscape, and the application of anti-IL-17A therapy under appropriate conditions could achieve a significant therapeutic effect, including in combination with ICIs therapy. However, further exploration of the accurate criteria for the application of anti-IL-17A in patients with MSS CRC is lacking in this study. Second, the heterogeneity of human tumors is much higher than that of the murine model; therefore, clinical evidence for the benefit of anti-IL-17A is needed. Excitingly, monoclonal antibodies against IL-17A are available. Secukinumab and Ixekizumab bind to IL-17A and achieved excellent therapeutic efficacy and safety in patients with psoriasis, psoriatic arthritis, and ankylosing spondylitis.^49 50^ Therefore, in a further clinical trial, it is necessary to fully implement this combination of anti-IL-17A and anti-PD-1 in the treatment of patients with MSS CRC.

## CONCLUSION

In summary, while targeting IL-17A is a promising clinical direction to sensitize MSS CRC to ICIs therapy, adequate preclinical and clinical research is still necessary. In this work, we used the combination of anti-IL-17A and anti-PD-1 therapy in the murine models of MSS CRC and observed a significant benefit. Mechanistic studies revealed that IL-17A upregulated PD-L1 expression via the IL-17A/P65/NRF1/miR-15b-5p axis at the post-transcriptional level in CRC cells, thereby suppressing the efficacy of immunotherapy. Meanwhile, the predictive value of *PD-L1* mRNA in prognosis and antitumor immunity in MSS CRC was preliminarily explored. Given its role in predicting CD8+ cell infiltration and overall survival, the *PD-L1* mRNA level might have potential as a biomarker to predict the efficiency of ICIs in MSS CRC; however, further work is needed to test this predictive value of *PD-L1* mRNA in clinical practice. Overall, our findings proved the feasibility of targeting IL-17A to sensitize the ICIs therapy in MSS CRC and revealed the novel mechanism by which IL-17A promotes PD-L1 expression in CRC cells for the first time.

## Data Availability

Data are available on reasonable request. All data relevant to the study are included in the article or uploaded as supplementary information. All data relevant to the study are included in the article or uploaded as supplementary information.

## References

[1] Siegel RL, Miller KD, Jemal A. Cancer statistics, 2020. CA Cancer J Clin 2020;70:7–30. 10.3322/caac.21590 31912902

[2] Patel SA, Minn AJ. Combination cancer therapy with immune checkpoint blockade: mechanisms and strategies. Immunity 2018;48:417–33. 10.1016/j.immuni.2018.03.007 29562193 PMC6948191

[3] Ganesh K, Stadler ZK, Cercek A, et al. Immunotherapy in colorectal cancer: rationale, challenges and potential. Nat Rev Gastroenterol Hepatol 2019;16:361–75. 10.1038/s41575-019-0126-x 30886395 PMC7295073

[4] Overman MJ, McDermott R, Leach JL, et al. Nivolumab in patients with metastatic DNA mismatch repair-deficient or microsatellite instability-high colorectal cancer (CheckMate 142): an open-label, multicentre, phase 2 study. Lancet Oncol 2017;18:1182–91. 10.1016/S1470-2045(17)30422-9 28734759 PMC6207072

[5] Le DT, Durham JN, Smith KN, et al. Mismatch repair deficiency predicts response of solid tumors to PD-1 blockade. Science 2017;357:409–13. 10.1126/science.aan6733 28596308 PMC5576142

[6] Fukuoka S, Hara H, Takahashi N, et al. Regorafenib plus nivolumab in patients with advanced gastric or colorectal cancer: an open-label, dose-escalation, and Dose-Expansion phase Ib trial (REGONIVO, EPOC1603). J Clin Oncol 2020;38:2053–61. 10.1200/JCO.19.03296 32343640

[7] Wilke CM, Wei S, Wang L, et al. Dual biological effects of the cytokines interleukin-10 and interferon-γ. Cancer Immunol Immunother 2011;60:1529–41. 10.1007/s00262-011-1104-5 21918895 PMC11029274

[8] Hersom M, Jørgensen JT. Companion and complementary Diagnostics-Focus on PD-L1 expression assays for PD-1/PD-L1 checkpoint inhibitors in non-small cell lung cancer. Ther Drug Monit 2018;40:9–16. 10.1097/FTD.0000000000000460 29084031

[9] Le DT, Uram JN, Wang H, et al. PD-1 blockade in tumors with mismatch-repair deficiency. N Engl J Med 2015;372:2509–20. 10.1056/NEJMoa1500596 26028255 PMC4481136

[10] Sun C, Mezzadra R, Schumacher TN. Regulation and function of the PD-L1 checkpoint. Immunity 2018;48:434–52. 10.1016/j.immuni.2018.03.014 29562194 PMC7116507

[11] Hsu J-M, Li C-W, Lai Y-J, et al. Posttranslational modifications of PD-L1 and their applications in cancer therapy. Cancer Res 2018;78:6349–53. 10.1158/0008-5472.CAN-18-1892 30442814 PMC6242346

[12] Duncan DJ, Scott M, Scorer P, et al. Assessment of PD-L1 mRNA and protein expression in non-small cell lung cancer, head and neck squamous cell carcinoma and urothelial carcinoma tissue specimens using RNAScope and immunohistochemistry. PLoS One 2019;14:e0215393. 10.1371/journal.pone.0215393 30986253 PMC6464208

[13] Ma L-J, Feng F-L, Dong L-Q, et al. Clinical significance of PD-1/PD-Ls gene amplification and overexpression in patients with hepatocellular carcinoma. Theranostics 2018;8:5690–702. 10.7150/thno.28742 30555574 PMC6276293

[14] Conroy JM, Pabla S, Nesline MK, et al. Next generation sequencing of PD-L1 for predicting response to immune checkpoint inhibitors. J Immunother Cancer 2019;7:18. 10.1186/s40425-018-0489-5 30678715 PMC6346512

[15] Tosolini M, Kirilovsky A, Mlecnik B, et al. Clinical impact of different classes of infiltrating T cytotoxic and helper cells (Th1, Th2, Treg, Th17) in patients with colorectal cancer. Cancer Res 2011;71:1263–71. 10.1158/0008-5472.CAN-10-2907 21303976

[16] Razi S, Baradaran Noveiry B, Keshavarz-Fathi M, et al. IL-17 and colorectal cancer: from carcinogenesis to treatment. Cytokine 2019;116:7–12. 10.1016/j.cyto.2018.12.021 30684916

[17] Wu P, Wu D, Ni C, et al. γδT17 cells promote the accumulation and expansion of myeloid-derived suppressor cells in human colorectal cancer. Immunity 2014;40:785–800. 10.1016/j.immuni.2014.03.013 24816404 PMC4716654

[18] Chen J, Ye X, Pitmon E, et al. IL-17 inhibits CXCL9/10-mediated recruitment of CD8^+^ cytotoxic T cells and regulatory T cells to colorectal tumors. J Immunother Cancer 2019;7:324. 10.1186/s40425-019-0757-z 31775909 PMC6880503

[19] Llosa NJ, Luber B, Tam AJ, et al. Intratumoral adaptive immunosuppression and type 17 immunity in mismatch repair proficient colorectal tumors. Clin Cancer Res 2019;25:5250–9. 10.1158/1078-0432.CCR-19-0114 31061070 PMC6726531

[20] Willis JA, Overman MJ, Vilar E. Mismatch repair-proficient colorectal cancer: finding the right time to respond. Clin Cancer Res 2019;25:5185–7. 10.1158/1078-0432.CCR-19-1447 31263028 PMC6726523

[21] Le Gouvello S, Bastuji-Garin S, Aloulou N, et al. High prevalence of FOXP3 and IL17 in MMR-proficient colorectal carcinomas. Gut 2008;57:772–9. 10.1136/gut.2007.123794 17965063

[22] Li W, Li H, Liu R, et al. Comprehensive analysis of the relationship between Ras and Raf mutations and MSI status of colorectal cancer in northeastern China. Cell Physiol Biochem 2018;50:1496–509. 10.1159/000494649 30359964

[23] Farlow SJ, Jerusalmi A, Sano T. Enhanced transduction of colonic cell lines in vitro and the inflamed colon in mice by viral vectors, derived from adeno-associated virus serotype 2, using virus-microbead conjugates bearing lectin. BMC Biotechnol 2007;7:83. 10.1186/1472-6750-7-83 18045466 PMC2217541

[24] Gao Y, Li X, Yang M, et al. Colitis-accelerated colorectal cancer and metabolic dysregulation in a mouse model. Carcinogenesis 2013;34:1861–9. 10.1093/carcin/bgt135 23615396

[25] Kettunen HL, Kettunen ASL, Rautonen NE. Intestinal immune responses in wild-type and ^ApcMin/+^ mouse, a model for colon cancer. Cancer Res 2003;63:5136–42. 12941845

[26] Chen B, Khodadoust MS, Liu CL, et al. Profiling tumor infiltrating immune cells with CIBERSORT. Methods Mol Biol 2018;1711:243–59. 10.1007/978-1-4939-7493-1_12 29344893 PMC5895181

[27] Lennerz JK, van der Sloot KWJ, Le LP, et al. Colorectal cancer in Crohn's colitis is comparable to sporadic colorectal cancer. Int J Colorectal Dis 2016;31:973–82. 10.1007/s00384-016-2574-x 27026089

[28] Baker A-M, Cross W, Curtius K, et al. Evolutionary history of human colitis-associated colorectal cancer. Gut 2019;68:985–95. 10.1136/gutjnl-2018-316191 29991641 PMC6580738

[29] Pekow J, Meckel K, Dougherty U, et al. miR-193A-3P is a key tumor suppressor in ulcerative colitis-associated colon cancer and promotes carcinogenesis through upregulation of IL17RD. Clin Cancer Res 2017;23:5281–91. 10.1158/1078-0432.CCR-17-0171 28600480 PMC5581687

[30] Kim CG, Jang M, Kim Y, et al. Vegf-A drives TOX-dependent T cell exhaustion in anti-PD-1-resistant microsatellite stable colorectal cancers. Sci Immunol 2019;4:eaay0555. 10.1126/sciimmunol.aay0555 31704735

[31] Knudson KM, Hicks KC, Alter S, et al. Mechanisms involved in IL-15 superagonist enhancement of anti-PD-L1 therapy. J Immunother Cancer 2019;7:82. 10.1186/s40425-019-0551-y 30898149 PMC6429734

[32] West NR, McCuaig S, Franchini F, et al. Emerging cytokine networks in colorectal cancer. Nat Rev Immunol 2015;15:615–29. 10.1038/nri3896 26358393

[33] Zhang J, Sun R, Liu J, et al. Reverse of NK cytolysis resistance of type II cytokine predominant-human tumor cells. Int Immunopharmacol 2006;6:1176–80. 10.1016/j.intimp.2006.02.011 16714222

[34] Blesa JR, Prieto-Ruiz JA, Abraham BA, et al. NRF-1 is the major transcription factor regulating the expression of the human TOMM34 gene. Biochem Cell Biol 2008;86:46–56. 10.1139/O07-151 18364745

[35] Nishino M, Ramaiya NH, Hatabu H, et al. Monitoring immune-checkpoint blockade: response evaluation and biomarker development. Nat Rev Clin Oncol 2017;14:655–68. 10.1038/nrclinonc.2017.88 28653677 PMC5650537

[36] Koch M, Beckhove P, Op den Winkel J, et al. Tumor infiltrating T lymphocytes in colorectal cancer: tumor-selective activation and cytotoxic activity in situ. Ann Surg 2006;244:986–93. 10.1097/01.sla.0000247058.43243.7b 17122624 PMC1856622

[37] Pagès F, Galon J, Dieu-Nosjean M-C, et al. Immune infiltration in human tumors: a prognostic factor that should not be ignored. Oncogene 2010;29:1093–102. 10.1038/onc.2009.416 19946335

[38] Mlecnik B, Van den Eynde M, Bindea G, et al. Comprehensive Intrametastatic immune quantification and major impact of immunoscore on survival. J Natl Cancer Inst 2018;110:djx123:97–108. 10.1093/jnci/djx123 28922789

[39] Chalabi M, Fanchi LF, Dijkstra KK, et al. Neoadjuvant immunotherapy leads to pathological responses in MMR-proficient and MMR-deficient early-stage colon cancers. Nat Med 2020;26:566–76. 10.1038/s41591-020-0805-8 32251400

[40] Kao SC, Cheng YY, Williams M, et al. Tumor Suppressor microRNAs Contribute to the Regulation of PD-L1 Expression in Malignant Pleural Mesothelioma. J Thorac Oncol 2017;12:1421–33. 10.1016/j.jtho.2017.05.024 28629895

[41] Ji D, Zhan T, Li M, et al. Enhancement of sensitivity to Chemo/Radiation therapy by using miR-15b against DCLK1 in colorectal cancer. Stem Cell Reports 2018;11:1506–22. 10.1016/j.stemcr.2018.10.015 30449704 PMC6294114

[42] Frampton AE, Krell J, Gall TMH, et al. miR-15b and miR-17 are tumor-derived plasma microRNAs dysregulated in colorectal neoplasia. Ann Surg 2015;262:e61–2. 10.1097/SLA.0000000000000605 24646542

[43] Sun L-N, Zhi Z, Chen L-Y, et al. SIRT1 suppresses colorectal cancer metastasis by transcriptional repression of miR-15b-5p. Cancer Lett 2017;409:104–15. 10.1016/j.canlet.2017.09.001 28923398

[44] Liu C, Yao Z, Wang J, et al. Macrophage-Derived CCL5 facilitates immune escape of colorectal cancer cells via the p65/STAT3-CSN5-PD-L1 pathway. Cell Death Differ 2020;27:1765–81. 10.1038/s41418-019-0460-0 31802034 PMC7244707

[45] Mezzadra R, Sun C, Jae LT, et al. Identification of CMTM6 and CMTM4 as PD-L1 protein regulators. Nature 2017;549:106–10. 10.1038/nature23669 28813410 PMC6333292

[46] Wei Y, Shi D, Liang Z, et al. IL-17A secreted from lymphatic endothelial cells promotes tumorigenesis by upregulation of PD-L1 in hepatoma stem cells. J Hepatol 2019;71:1206–15. 10.1016/j.jhep.2019.08.034 31499129

[47] Aotsuka A, Matsumoto Y, Arimoto T, et al. Interleukin-17 is associated with expression of programmed cell death 1 ligand 1 in ovarian carcinoma. Cancer Sci 2019;110:3068–78. 10.1111/cas.14174 31432577 PMC6778630

[48] Amicarella F, Muraro MG, Hirt C, et al. Dual role of tumour-infiltrating T helper 17 cells in human colorectal cancer. Gut 2017;66:692–704. 10.1136/gutjnl-2015-310016 26719303 PMC5529969

[49] Blauvelt A. Ixekizumab: a new anti-IL-17A monoclonal antibody therapy for moderate-to severe plaque psoriasis. Expert Opin Biol Ther 2016;16:255–63. 10.1517/14712598.2016.1132695 26666707

[50] Esfahani K, Miller WH. Reversal of autoimmune toxicity and loss of tumor response by interleukin-17 blockade. N Engl J Med 2017;376:1989–91. 10.1056/NEJMc1703047 28514612

