# Correction: Blocking IL-17A enhances tumor response to anti-PD-1 immunotherapy in microsatellite stable colorectal cancer

**DOI:** 10.1136/jitc-2020-001895corr1

**Published:** 2021-10-13

Liu C, Liu R, Wang B, *et al*. Blocking IL-17A enhances tumor response to anti-PD-1 immunotherapy in microsatellite stable colorectal cancer. *J Immunother Cancer.* 2021;9:e001895. doi: 10.1136/jitc-2020-001895.

This article has been corrected since it first published. The provenance and peer review statement has been added.

