# Supplementary material for: Blocking IL-17A enhances tumor response to anti-PD-1 immunotherapy in microsatellite stable colorectal cancer
Source: J Immunother Cancer. 2021 Jan 17;9(1):e001895. doi: 10.1136/jitc-2020-001895 (PMC7813395; doi:10.1136/jitc-2020-001895)
Supplement: Supplementary data [file jitc-2020-001895supp001.pdf]

SUPPLEMENTARY METHODS

Reagents, Antibodies, and Plasmids

Murine IL-2, IL-17A, IL6, TNFα, IL-8, IL-10, IL-21, IFN-γ and anti-CD3 antibody, human IL-17A were purchased from PeproTech (NJ, USA). Bay11-7082 were purchased from Selleck (Texas, USA). Azoxymethane (AOM) was purchased from Sigma Aldrich (MO, USA). Dextran sodium sulfate (DSS) was purchased from MPBIO (CA, USA). The antibodies used in the current study are listed at the supplemental table 2. Mouse NRF1 and YY1 over-expression vectors and knockdown vectors were obtained from Vigenebio (Jinan, China). pGL-4.20 and pMIR-reporter plasmids were gifts from School of Life Science and Technology, Harbin Institute of Technology. The shRNA sequences of NRF1 is 5'-TATCCGGAAGAGGCAACAAACCTCGAG-3' and 5'-CCGTTGCCCAAGTGAATTATT-3'. The shRNA sequences of YY1 is 5'-CGATGGTTGTAATAAGAAGTT-3' and 5'- GACGACGACTACATTGAACAA -3'.

In situ hybridization (ISH) and immunohistochemical staining (IHC)

ISH were performed according to the manufacturer's instruction of the HOTAIR and microRNA ISH Optimization Kits (Boster, Wuhan, China). The triple digoxigenin-labeled antisense locked nucleic acid (LNA)-modified probes for PD-L1 and miR-15b-5p were synthesized by Boster Biotech (Wuhan, China).

IHC were performed as previously described. In brief, Paraffin-embedded 4-μm-thick sections were deparaffinized, heated in citrate buffer (0.01 M), treated with 0.3% H_2O_2(v/v), and re-hydrated. After blocking, the sections were incubated with antibody in a humid chamber at 4°C overnight. After several rinses in Phosphate-Buffered Saline

(PBS), the sections were incubated in the biotinylated secondary antibody. Subsequently, the slides were rinsed in PBS, exposed to diaminobenzidine, and counterstained with Hematoxylin. After serial dehydration, the slides were mounted for microscopic examination. As a negative control for the staining procedure, the primary antibody was omitted. All antibody information is available at supplementary table 2.

Evaluation of IHC and ISH variables

The procedure for evaluation of immunohistochemical variables PD-L1(Protein), NRF1 and ISH variables miR-15b-5p and PD-L1(mRNA) were established as previous described. Briefly, the intensity staining was scored as 0 (absent), 1 (weak), 2 (moderate), and 3 (marked). Percentage scores were assigned by the percentage of stained cells in a chosen field, as 1, 0–25%; 2, 26–50%; 3, 51–75%; and 4, 76–100%. The scores of each tumor sample were multiplied to give a final score of 0–14. PD-L1 was evaluated based on immunostaining in tumor cells. The expression intensity was scored as 0 (absent), 1 (weak), 2 (moderate) or 3 (strong).

IL-17A+, CD3+, CD8+ and CD33+ cells variables based on the following method. At a low-power field (×100), the tissue sections were screened, and the 5 most representative fields were selected. Thereafter, respective areas were measured at ×400 magnification. The numbers of nucleated stromal cells in the tumor regions were then counted manually and expressed as cells per field. Positively stained cells that were smaller than the size of circulating T cells (10 μm) were excluded from counting. All analysis was performed by 2 independent observers who were blinded to the clinical outcome. The average of counts by 2 investigators was applied in the following analysis

to minimize interobserver variability.

Western Blotting

The cells or tissues were washed with ice-cold PBS and lysed with RIPA buffer containing protease inhibitors. The whole cell lysates were electrophoresed in 12% SDS-PAGE and then transferred to polyvinylidene fluoride (PVDF) membranes. The membranes were blocked with 5% nonfat milk, and then incubated with a specific antibody overnight at 4°C. After incubation with primary antibodies at 4°C overnight, membranes were incubated with horseradish peroxidase-conjugated secondary antibodies for 1 hour at RT. ECL was used to visualize the immunoblot signals. The Abs used are listed in Supplementary Table 2.

Real Time-quantitative PCR (RT-qPCR)

Total RNA was extracted with TRIzol(Thermo Fisher Scientific, Waltham, USA), and 1 μg RNA was used for reverse transcription into complementary DNA (cDNA) using the PrimeScript RT-PCR Kit (Takara, Tokyo, Japan). Real-time polymerase chain reaction (PCR) was conducted using the SYBR Premix Ex-Taq II kit (Takara, Tokyo, Japan) on the Quant Studio 3 Real-Time PCR System (Applied Biosystems, CA, USA). The fold changes were determined using the 2-△△CT method. All primer information is available at Supplementary Table 3.

Flow cytometry (FACS)

CT26 and MC38 cells were stained with PD-L1-PE Abs according to the manufacturers' instructions. Immunophenotypic analyses of tumor infiltrating lymphocytes were assessed by flow cytometry. Abs to stain for MDSCs were CD11b-PE, and Gr1-FITC.

Liu C, *et al. J Immunother Cancer* 2021; 9:e001895. doi: 10.1136/jitc-2020-001895

For cytotoxic lymphocyte cell activation markers, cells were stained with Ab specific for CD3-FITC, CD8-PE and IFN-γ-PE-dazzle. The lymphocytes isolated from the tumor tissue were pretreated with Fixation & Permeabilization Kit (BD product). Next cells were incubated on ice for 30 min, washed and fixed in PBS containing 1% formalin for flow cytometric analysis on a LSRII flow cytometer. The Abs used are listed in Supplementary Table2.

Immunofluorescence analysis (IF)

Mice were euthanized by carbon dioxide and the flank tumors were resected at 24 days post-tumor injection. Tumors were fixed in 4% paraformaldehyde in PBS overnight at 4ºC, and then placed in 15% sucrose solution for 1 hour and in 30% sucrose solution overnight. Tissue was embedded in tissue freezing medium, sectioned, and stained by DAPI and antibodies against CD3, CD11b. For immunofluorescence analysis, tissues were stained with CD3 antibody, CD11b antibody followed by Alexa Fluor Plus 488 conjugated goat anti-rabbit IgG, Alexa Fluor 647 conjugated goat anti-rat IgG. Images were acquired on a Zeiss 710 Meta multi-photon confocal microscope.

Chromatin immunoprecipitation (Ch-IP)

CT26 and MC38 (1×10^8 cells) were crosslinked with 4% paraformaldehyde at 37 °C for 15 min. After the cells were washed with PBS, they were resuspended in 600 μL of ChIP lysis buffer (50 mM Tris (pH 8.0), 1% SDS, 10mM EDT and 1 mM PMSF). The DNA was sheared to lengths between 200 bp and 1000 bp by sonication. The protein-DNA complexes were precipitated with a ChIP-grade-NRF1 or ChIP-grade-YY1 antibody, with normal IgG antibody serving as a negative control, overnight at 4 °C.

Protein A/G magnetic beads were used to purify the complexes, and the cross-linkages were reversed at 65 °C for 3 h. Next, the DNA was purified using a PCR Purification Kit (Qiagen, CA, USA). Finally, the binding capacity between NRF1 and miR-15b-5p promoter or YY1 and miR-15b-5p promoter was detected PCR. The miR-15b-5p promoter primer sequences used in the Ch-IP-PCR assay were listed in Supplementary Table 3.

Dual luciferase activity assay

The 3´-UTR of mice and human PD-L1 cDNA containing the putative target site for the miR-15b-5p was inserted into the pMIR-Reporter-control vector (Promega, WI, USA) immediately downstream of the luciferase gene. CT26 or MC38 cells were transfected with reporter plasmids and pRL-TK (Promega), together with 100 nM of miR-15b-5p mimics or NCs utilizing Lipofectamine 2000 (Invitrogen, CA, USA) according to the manufacturer's protocol, respectively. miR-15b-5p promoter construct contained the wild-type or mutant NRF1-binding site in pGL4.2-Luc plasmids. CT26 and MC38 cells were transfected with pGL4.2-Luc plasmids, pRL-TK and pNRF1 or shNRF1 plasmids. After transfection for 48h, cells were lysed by lysis buffer. Luciferase activity was measured utilizing the Dual Luciferase Reporter Assay System (Promega). Firefly luciferase activity was normalized to Renilla luciferase activity for each transfected sample.

Liu C, *et al. J Immunother Cancer* 2021; 9:e001895. doi: 10.1136/jitc-2020-001895
