# Supplementary material for: Blocking IL-17A enhances tumor response to anti-PD-1 immunotherapy in microsatellite stable colorectal cancer
Source: J Immunother Cancer. 2021 Jan 17;9(1):e001895. doi: 10.1136/jitc-2020-001895 (PMC7813395; doi:10.1136/jitc-2020-001895)
Supplement: Supplementary data [file jitc-2020-001895supp002.pdf]

Supplemental Table 1. Clinical characteristics of the 261 CRC patients.

**Category**	**N (%)**
Gender	
Male	155 (59.4)
Female	106 (40.6)
Age	
<60	109 (41.8)
>60	152 (58.2)
Histological differentiation	
Poorly	48 (18.4)
Moderately	196 (75.1)
Well	17 (6.5)
Depth of invasion	
T1	2 (0.8)
T2	16 (6.1)
T3	216 (82.8)
T4	27 (10.3)
Lymph node metastasis	
N0	203 (77.8)
N1	47 (18)
N2	11 (4.2)
Metastasis	
M0	256 (98.1)
M1	5 (1.9)
Pathologic stage	
Ⅰ	18 (6.9)
Ⅱ	182 (69.7)
Ⅲ	56 (21.5)
Ⅳ	5 (1.9)
MSI status	
MSS	210 (80.5)
MSI-H	51 (19.5)

Liu C, *et al. J Immunother Cancer* 2021; 9:e001895. doi: 10.1136/jitc-2020-001895

Supplemental Table 2. Antibodies used in experiments.

Antibody	Supplier, Clone	Application
PD-L1	eBioscience, MIH1	WB, IHC
NRF1	CST, D9K6P	WB, ChIP
P65	CST, D14E12	WB
pP65	CST, 93H1	WB
YY1	CST, D5D9Z	WB, ChIP
Beta-actin	Proteintech, 7D2C10	WB
CD3	Alfetronic, UMAB54	IHC
CD8	Alfetronic, SP16	IHC
CD33	Alfetronic, PWS44	IHC
IL-17A	RD, 41809	IHC
MLH1	BD, G168-15	IHC
MSH2	BD, G219-1129	IHC
MSH6	Abcam, EPR3945	IHC
PMS2	Abcam, EPR3947	IHC
NRF1	Abcam, EPR5554(N)	IHC
PD-L1	Biolegend, MIH7	FACS
CD3	Biolegend, 17A2	FACS
CD8	Biolegend, 53-6.7	FACS
IFNγ	Biolegend, XMG1.2	FACS
CD11b	Biolegend, M1/70	FACS
Gr1	Biolegend, RB6-8C5	FACS
CD3	Abcam, PS1	IF
CD11b	Abcam, EP1345Y	IF
IgG	Bio X Cell, MOPC-21	mouse experiments
IL-17A	Bio X Cell, 17F3	mouse experiments
PD-1	Bio X Cell, RMP1-14	mouse experiments

Supplemental Table 3. Primers for PCR.

Application	Gene		Sequences
Quantitative	**m-PD-L1**	Forward	GCTCCAAAGGACTTGTACGTG
		Reverse	TGATCTGAAGGGCAGCATTTC
	**m-Bactin**	Forward	GGCTGTATTCCCCTCCATCG
		Reverse	CCAGTTGGTAACAATGCCATGT
	**h-PD-L1**	Forward	TGGCATTTGCTGAACGCATTT
		Reverse	TGCAGCCAGGTCTAATTGTTTT
	**h-Bactin**	Forward	CATGTACGTTGCTATCCAGGC
		Reverse	CTCCTTAATGTCACGCACGAT
	**miR-15b-5p**	Forward	TAGCAGCACATCATGGTT
		Reverse	GTCGTATCCAGTGCAGGGTCCGAGGT
	**h-U6**	Forward	CTCGCTTCGGCAGCACATATACT
		Reverse	ACGCTTCACGAATTTGCGTGTC
Reverse Transcription	**miR-15b-5p**	GTCGTATCCAGTGCAGGGTCCGAGGTATTCGCACT GGATACGACAACCAT	
	**U6**	AAAATATGGAACGCTTCACGAATTTG	
Ch-IP	**m-NRF1**	Forward	CGTAGCCCTGCTTGCTCT
		Reverse	CGCCTAAAATCCAAACTCG
	**m-YY1**	Forward	GAATTTCCGTTCGAGTTTGG
		Reverse	TGTCGGCGGGTCCACT
	**h-NRF1**	Forward	AATCCCTCGCTCTTCC
		Reverse	CGCCTAAAATACAAACTCG

Liu C, *et al. J Immunother Cancer* 2021; 9:e001895. doi: 10.1136/jitc-2020-001895
