# Supplementary figures and images for: Blocking IL-17A enhances tumor response to anti-PD-1 immunotherapy in microsatellite stable colorectal cancer
Source: J Immunother Cancer. 2021 Jan 17;9(1):e001895. doi: 10.1136/jitc-2020-001895 (PMC7813395; doi:10.1136/jitc-2020-001895)

Figure S1

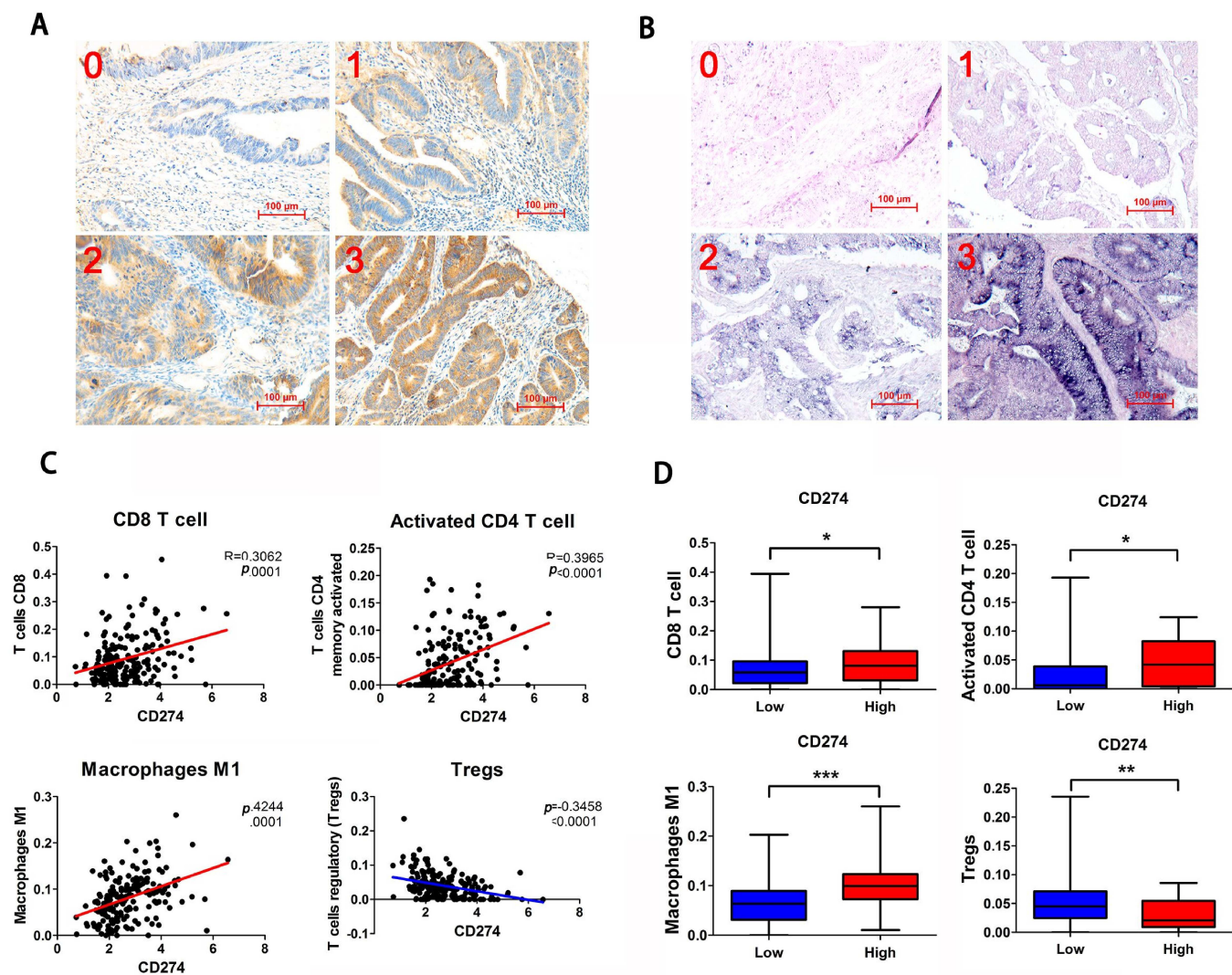

Supplement: Supplementary data [file jitc-2020-001895supp003.pdf]

Figure S2

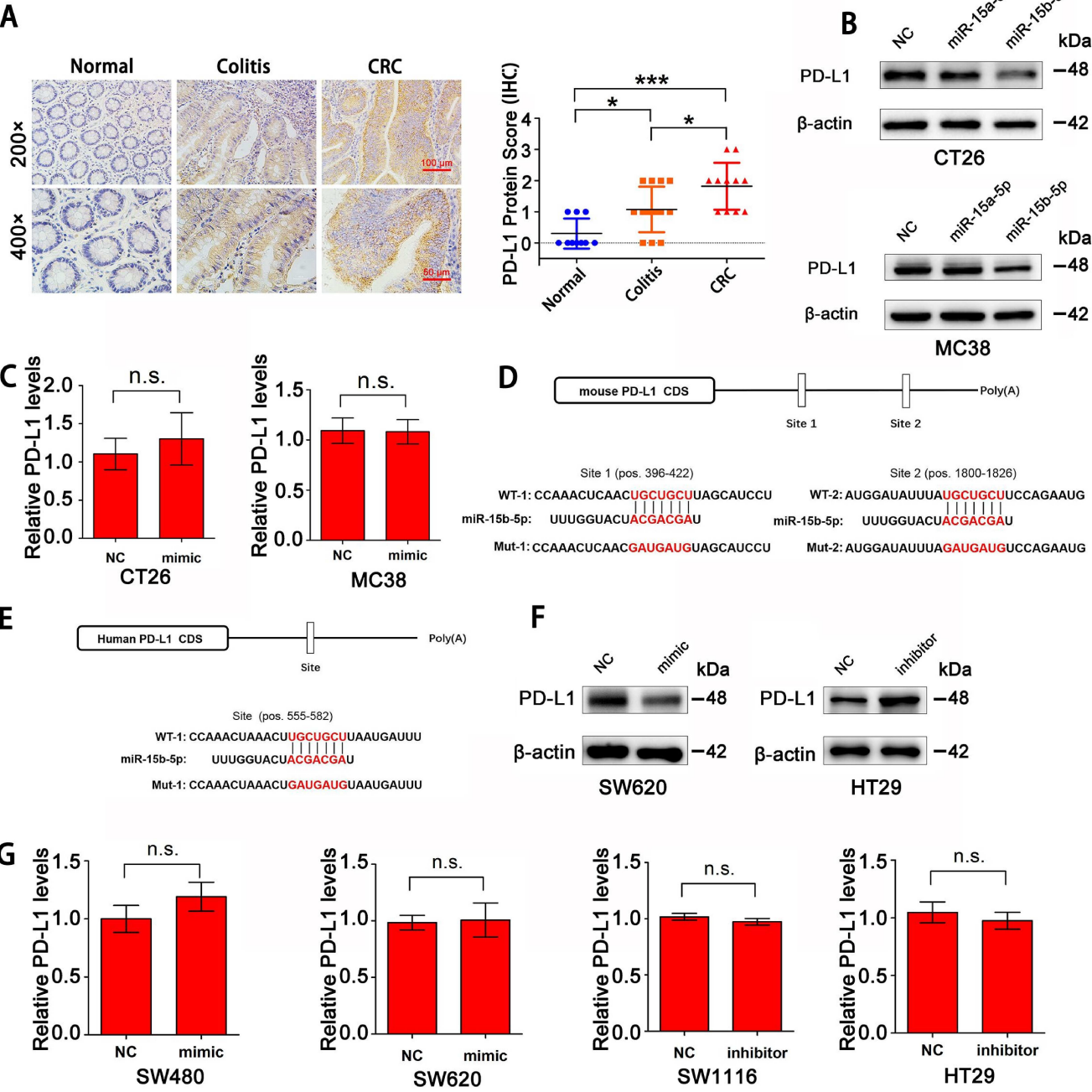

Supplement: Supplementary data [file jitc-2020-001895supp004.pdf]

Figure S3

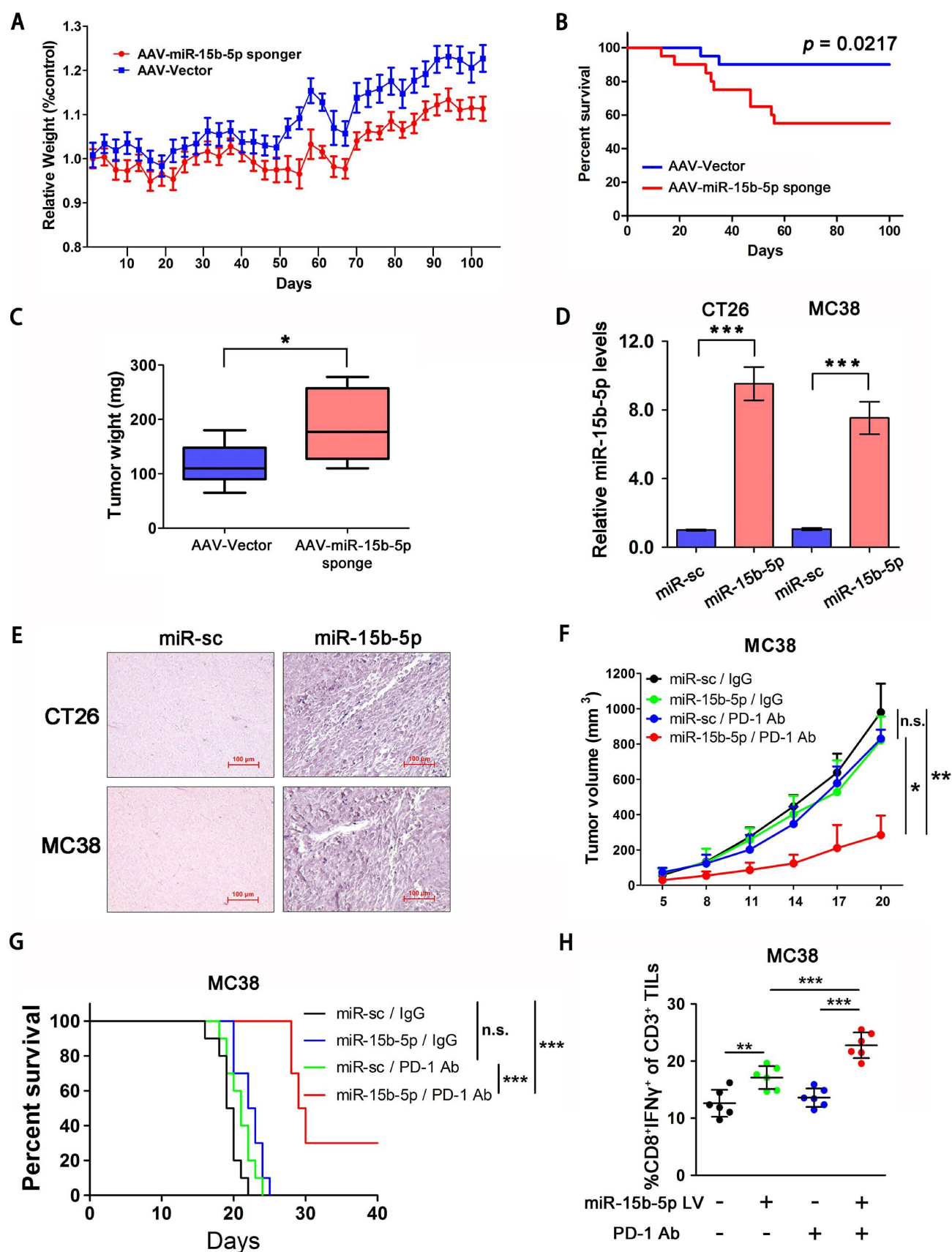

Supplement: Supplementary data [file jitc-2020-001895supp005.pdf]

Figure S4

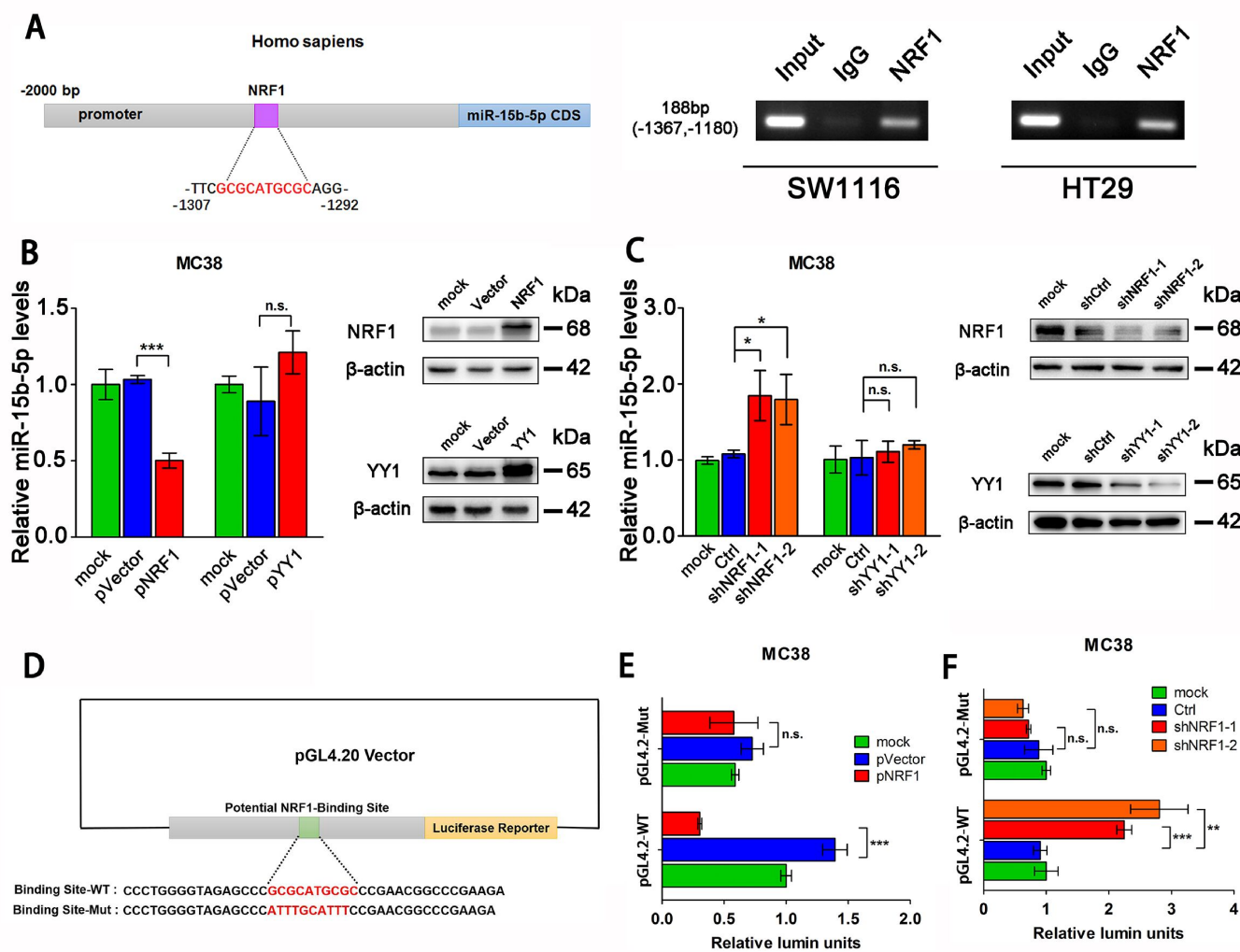

Supplement: Supplementary data [file jitc-2020-001895supp006.pdf]

Figure S5

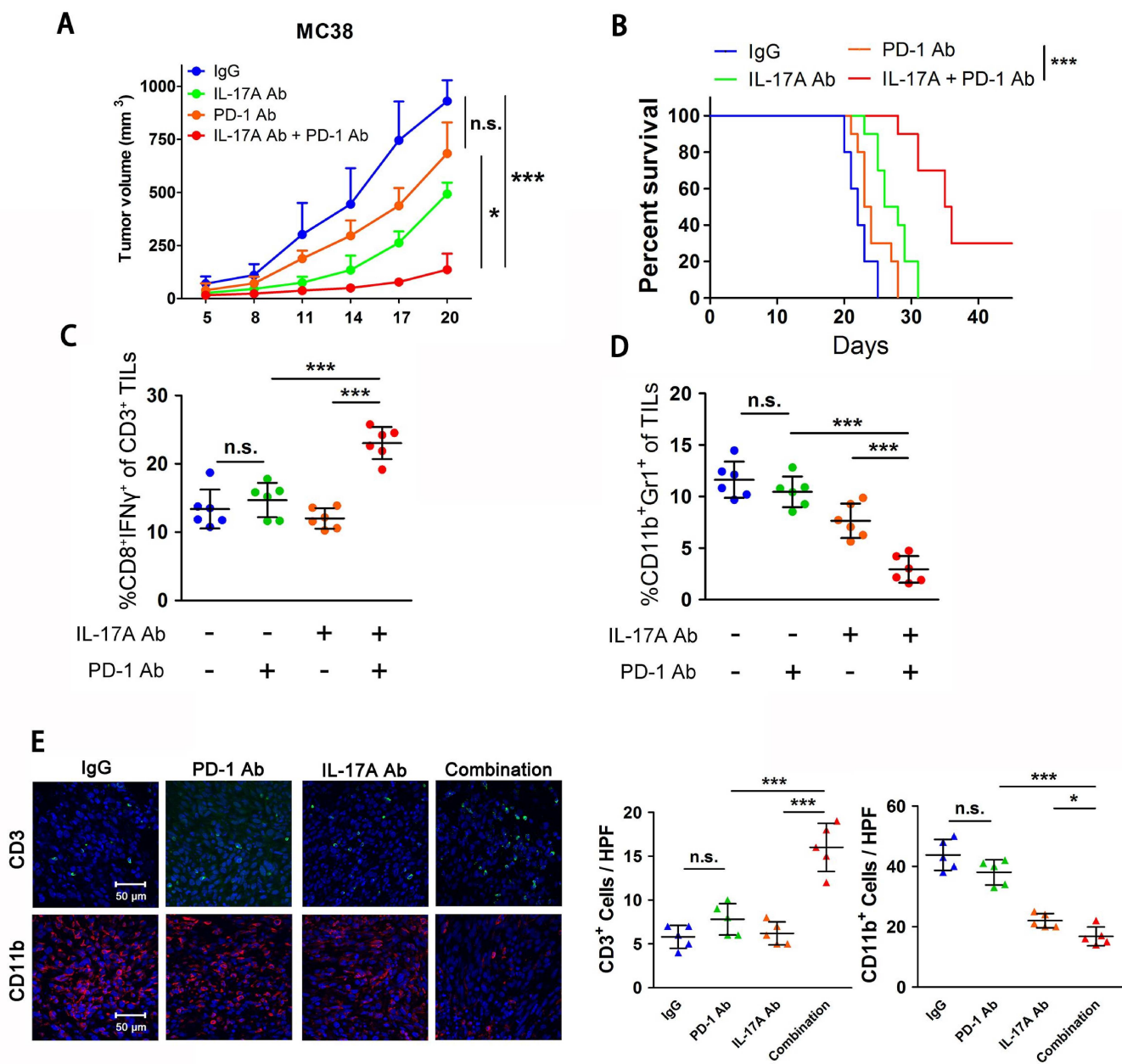

Supplement: Supplementary data [file jitc-2020-001895supp007.pdf]
