# Supplementary material for: Blocking IL-17A enhances tumor response to anti-PD-1 immunotherapy in microsatellite stable colorectal cancer
Source: J Immunother Cancer. 2021 Jan 17;9(1):e001895. doi: 10.1136/jitc-2020-001895 (PMC7813395; doi:10.1136/jitc-2020-001895)
Supplement: Supplementary data [file jitc-2020-001895supp008.pdf]

SUPPLEMENTARY FIGURE LEGENDS

Supplementary Figure 1 PD-L1 mRNA level is associated with CD8+ T cells infiltration and prognosis in MSS CRC. (A) PD-L1 protein expression in CRC tissues as determined by IHC (scale bar, 100 μm). (B) PD-L1 mRNA level in CRC tissues as determined by ISH (scale bar, 100 μm). (C) CIBERSORT analyzes the correlation between CD274 and CD8+ T cells, activated CD4 T cells, Macrophages M1 and Tregs in CRC samples of TCGA database. (D) CIBERSORT analyzes the correlation between CD274 and CD8+ T cells, activated CD4 T cells, Macrophages M1 and Tregs in MSS CRC samples of TCGA database. *P* values and R values were calculated based on the analysis of Pearson’s correlation. Student’s t test. *p<0.05, **p<0.01 and ***p<0.001.

Supplementary Figure 2 miR-15b-5p is a regulator of PD-L1 at post-transcriptional level in MSS CRC. (A) PD-L1 expression in normal colon epithelial cells, inflammation-associated colon epithelial cells and cancer cells of colitis-associated cancer patients (scale bar, 200 μm; magnification scale bar, 100 μm). (B) miR-15b-5p, but not miR-15a-5p, inhibited PD-L1 expression in mouse intestinal cancer cell lines CT26 and MC38, but not miR-15a-5p. (C) RT-qPCR were performed to analysis the expression of PD-L1 in mRNA level after transfection of miR-15b-5p mimic in CT26 and MC38 cell lines. (D) Schematic representation of sequences of the wild-type and the mutated 3′-UTR luciferase reporters of mice. (E) Schematic representation of sequences of the wild-type and the mutated 3′-UTR luciferase reporters of human. (F) Expression of PD-L1 in protein levels were analyzed by

Western Blotting analysis after transfection of miR-15b-5p mimic or inhibitor into SW620 and HT29 cell lines. (G) RT-qPCR were performed to analysis the expression of PD-L1 in mRNA level after transfection of miR-15b-5p mimic or inhibitor in human CRC cell lines. Data represent mean ± SD. Results are representative of at least 3 separate experiments. Student's t test. *p<0.05, **p<0.01 and ***p<0.001.

Supplementary Figure 3 miR-15b-5p inhibits CRC tumorigenesis and sensitizes anti-PD-1 therapy by targeting PD-L1 in murine models. (A) Body weight changes of miR-15b-5p blocking and control mice were recorded throughout experiment. (B) Survival of mice miR-15b-5p blocking and control mice. (C) The weight of miR-15b-5p blocking and control group tumors were measured. (D) CT26 and MC38 cells were transfected with miR-15b-5p or miR-sc. miR-15b-5p mRNA levels were determined via RT-qPCR assay. (E) ISH analysis of miR-15b-5p expression in CT26 and MC38 tumors of subcutaneous transplantation. (F) Tumor growth of miR-sc, miR-15b-5p MC38 cells with or not PD-1 antibody treated in C57BL/6 mice (n = 7 mice per group). (G) Survival of mice bearing syngeneic MC38 tumors following treatment with PD-1 antibody. (n = 9 mice per group). (H) Intracellular cytokine staining of CD8+ IFN-γ + cells in the CD3+ T cell populations from isolated tumor-infiltrating lymphocytes. Data represent mean ± SD. ANOVA followed by Tukey's multiple comparison test was applied. Cumulative survival time was estimated by the Kaplan-Meier method, and the log-rank test was applied to compare the groups. *p<0.05, **p<0.01 and ***p<0.001.

Supplementary Figure 4 NRF1 is the major transcription factor for IL-17A to accumulate PD-L1 protein. (A) Schematic representation of predicted NRF1 binding sites within the human miR-15b-5p promoter (Left). Ch-IP analyses of NRF1 binding to the miR-15b-5p promoter using antibodies against NRF1 (Right). (B, C) Effects of NRF1/YY1 overexpression or knockdown on miR-15b-5p expression through RT-qPCR analyses of miR-15b-5p expression in MC38 cells. (D) Schematic representation of sequences of the wild-type and the mutated promoter luciferase reporters of miR-15b-5p in vector of pGL4.20. (E, F) Modulation of the miR-15b-5p promoter activity by NRF1 overexpression or knockdown in MC38 cells. Data represent mean ± SD. Results are representative of at least 3 separate experiments. Student's t test. *p<0.05, **p<0.01 and ***p<0.001.

Supplementary Figure 5 Blocking IL-17A enhances the efficacy of anti-PD-1 therapy in murine model by promoting PD-L1 protein degradation. (A) Tumor growth of MC38 cells with IL-17A antibody and/or PD-1 antibody treated in C57BL/6 mice (n = 7 mice per group). (B) Survival of mice bearing syngeneic MC38 tumors following treatment with IL-17A antibody and/or PD-1 antibody (n = 9 mice per group). (C) FACS analysis of staining of CD8+ IFN-γ+ cells in the CD3+ T cell populations from isolated tumor-infiltrating lymphocytes. (D) FACS analysis of staining of CD11b+ and Gr1+ cells in the tumor-infiltrating lymphocyte. (E) Immunofluorescence was used to analyze the staining of CD3+ cells and CD11b+ cells in MC38 tumors. Data represent mean ± SD. ANOVA followed by Tukey's multiple comparison test was applied.

Cumulative survival time was estimated by the Kaplan-Meier method, and the log-rank test was applied to compare the groups. *$p<0.05$, **$p<0.01$ and ***$p<0.001$.
